# Genetic Diversity and Population Structure of *Miscanthus sinensis* Germplasm in China

**DOI:** 10.1371/journal.pone.0075672

**Published:** 2013-10-07

**Authors:** Hua Zhao, Bo Wang, Junrong He, Junpin Yang, Lei Pan, Dongfa Sun, Junhua Peng

**Affiliations:** 1 College of Plant Science & Technology, Huazhong Agricultural University, Wuhan, Hubei, China; 2 Wuhan Botanical Garden, Chinese Academy of Sciences, Wuhan, Hubei, China; 3 Graduate University of Chinese Academy of Sciences, Beijing, China; 4 Institute of Horticulture, Sichuan Academy of Agricultural Sciences, Chengdu, China; 5 Crop Research Institute, Sichuan Academy of Agricultural Sciences, Chengdu, China; National Rice Research Center, United States of America

## Abstract

*Miscanthus* is a perennial rhizomatous C4 grass native to East Asia. Endowed with great biomass yield, high ligno-cellulose composition, efficient use of radiation, nutrient and water, as well as tolerance to stress, *Miscanthus* has great potential as an excellent bioenergy crop. Despite of the high potential for biomass production of the allotriploid hybrid *M*. ×*giganteus*, derived from *M*. *sacchariflorus* and *M. sinensis*, other options need to be explored to improve the narrow genetic base of *M*. *×giganteus*, and also to exploit other *Miscanthus* species, including *M*. *sinensis* (2n = 2x = 38), as bioenergy crops. In the present study, a large number of 459 *M. sinensis* accessions, collected from the wide geographical distribution regions in China, were genotyped using 23 SSR markers transferable from *Brachypodium distachyon*. Genetic diversity and population structure were assessed. High genetic diversity and differentiation of the germplasm were observed, with 115 alleles in total, a polymorphic rate of 0.77, Nei’s genetic diversity index (*He*) of 0.32 and polymorphism information content (PIC) of 0.26. Clustering of germplasm accessions was primarily in agreement with the natural geographic distribution. AMOVA and genetic distance analyses confirmed the genetic differentiation in the *M*. *sinensis* germplasm and it was grouped into five clusters or subpopulations. Significant genetic variation among subpopulations indicated obvious genetic differentiation in the collections, but within-subpopulation variation (83%) was substantially greater than the between-subpopulation variation (17%). Considerable phenotypic variation was observed for multiple traits among 300 *M*. *sinensis* accessions. Nine SSR markers were found to be associated with heading date and biomass yield. The diverse Chinese *M. sinensis* germplasm and newly identified SSR markers were proved to be valuable for breeding *Miscanthus* varieties with desired bioenergy traits.

## Introduction

*Miscanthus* (Gramineae) is a genus of rhizomatous perennial C4 grass. The features of rapid growth, efficient use of radiation, water and nutrient, and strong tolerance to biotic and abiotic stresses make *Miscanthus* an attractive biofuel crop. Intensive researches done in Europe and USA proved the widely cultivated species of *M.* × *giganteus* a dedicated energy crop [1]–[5], due to its enormous yield of aboveground standing biomass [6]–[7] and high content of ligno-cellulose [8]. However, *M*. × *giganteus* is a sterile triploid hybrid from a spontaneous cross between *M*. *sacchariflorus* and *M*. *senensis*. The sterility of *M.* × *giganteus* make it difficult to genetically improve the species and result in the narrow genetic basis that reduce climatic adaptation and overwintering survival in some extreme conditions [9]–[12]. *M. sinensis*, originated from the tropical and subtropical regions of Asia and the progenitor of *M*. × *giganteus*, is one of the most important members in *Miscanthus* genus [10]–[11]. Furthermore, biomass yield of *M. sinensis* might be better than *M. ×giganteus* in some places [13]–[14]. Therefore, it is necessary to develop other species of *Miscanthus* genus, including *M*. *sinensis*, as bioenergy crops, or to enrich the genetic diversity of *M.* × *giganteus* using *M*. *sinensis* germplasm.

As one parent of *M*. *×giganteus*, *M*. *sinensis* can be used not only as a genetic resource for development of new hybrids or improvement of fertile lines, but also as an alternative biofuel crop with high potential in biomass production based on a series of studies on agronomy, productivity and utilization [15]–[18]. The *M. sinensis* germplasm shows rich morphological variability related with the distinct original environments. For development of new *Miscanthus* varieties, understanding of the genetic diversity and target traits of the *M*. *sinensis* germplasm is necessary. Intensive studies on *Miscanthus* in the past mainly focused on the biomass potential of *M*. *×giganteus*. The studies conducted in *M*. *sinensis* included genetic map construction, QTL mapping and genetic diversity evaluation using a limited number of *M*. *sinensis* germplasm accessions [19]–[20] and molecular markers, mainly random amplified polymorphic DNA (RAPD) and amplified fragment length polymorphism (AFLP) [21]–[23]. To date, a limited number of SSR markers have been developed for *Miscanthus* [24]–[26]. The most recent studies reported a linkage map consisting of single nucleotide polymorphism(SNP)markers in *M*. *sinensis* [27].

China is the major distribution area or diversity center of *M*. *sinensis* [28]. This species is widely distributed in China, covering distinct ecological environments such as river bands, abandoned lands, foothills and mountains. During the long-term evolution process, *M*. *sinensis* has accumulated a broad range of genetic variation for its adaptation to various environmental conditions in China. The diverse pool of *M*. *sinensis* germplasm can be exploited to improve its tolerance to biotic and abiotic stresses or to optimize properties for cellulosic ethanol production.

Transferable SSR markers are practical and efficient for genetic diversity evaluation of *Miscanthus* [29]–[30]. Although more and more genetic studies have been conducted on *M*. *sinensis* [5], little is known of the genetic diversity of Chinese *M*. *sinensis* germplasm. In the present study, a large collection of 459 Chinese *M*. *sinensis* accessions, covering all of the distribution areas in China, was adopted. The *M*. *sinensis* collections were genotyped using 23 SSR primer pairs developed based on the genome sequence of *Brachypodium distachyon* [30], and phenotyped for three bioenergy-related traits, biomass yield, heading date and plant height. Genetic diversity was evaluated, population structure was assessed, and trait-marker association was analyzed in this diverse panel of Chinese *M*. *sinensis* germplasm.

## Results

### Profile of the Transferable SSR Markers in *M*. *sinensis*

The 23 Brachypodium SSR primer pairs detected 115 alleles in a panel of 459 *M*. *sinensis* accessions. Among the 115 alleles, 16 were the rare and 90 were the frequent alleles. On the average, 4.3 alleles were detected per SSR primer pair, with a range of 1–16. For the germplasm panel, the rate of polymorphic alleles, Nei’s genetic diversity (*He*) and polymorphism information content (PIC) were 0.77, 0.32 and 0.26, respectively (Table 1). Some alleles were present and absent in one or two groups. There were 21 alleles absent in one or two groups, such as DBM4-2, DBM29-4, DBM39-2 and DBM51-1 etc. Also, the unique alleles DBM38-1 and DBM38-3 were detected only in Group 2 and DBM58-5 was only found in Group 1. Thus this study presented a sizeable molecular marker dataset in a diverse panel of *M. sinensis* germplasm with high diversity and some rare and group-unique alleles (Table 1, Table S1).

**Table 1:** Genetic diversity for each subpopulation grouped by structure analysis.

Group	G1	G2	G3	G4	G5	Overall
Sample size	92	94	96	86	91	459
Major allele frequency	0.80	0.81	0.79	0.81	0.81	0.77
Genetic diversity	0.27	0.26	0.28	0.25	0.26	0.32
PIC^a^	0.23	0.22	0.23	0.21	0.21	0.26

a Polymorphism information content.

### Population Structure and Genetic Diversity

**Figure 1:**
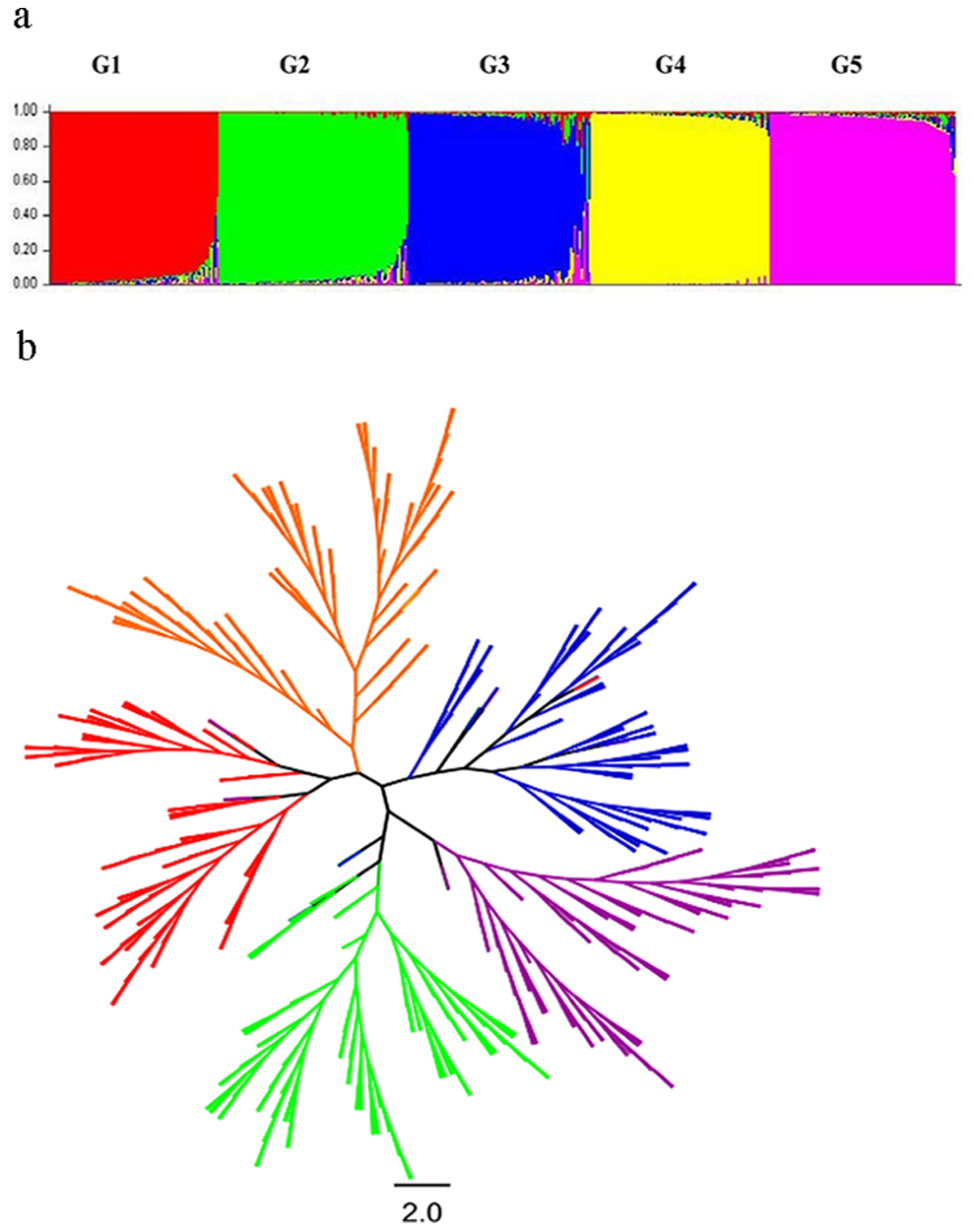
Molecular classification of Chinese *M*. *sinensis* germplasm. a Population structure of Chinese *M*. *sinensis* germplasm. b Classification of Chinese *M*. *sinensis* germplasm based on neighbor-joining tree clustering analysis. Note: The colors of the branch is consistent with the colors of structure group in a (G1 = *red*, G2 = *green*, G3 = *blue*, G4 = *yellow*, and G5 = *purple*). The vertical bars represent each genotype while division of the bars shows the genome content based on the sub-population identity.

The population structure was analyzed on the basis of SSR marker data using the model-based software STRUCTURE [31] to identify the main clusters with genetic similarity within the *M*. *sinensis* groups. The structure analysis was performed by setting the range of possible number of subpopulations (k) as 1 to 10. Based on an Evano test, the result showed that 5 was the optimum k number of subpopulations, indicating at least five distinct groups in the *M. sinensis* panel (Fig. 1).

**Figure 2:**
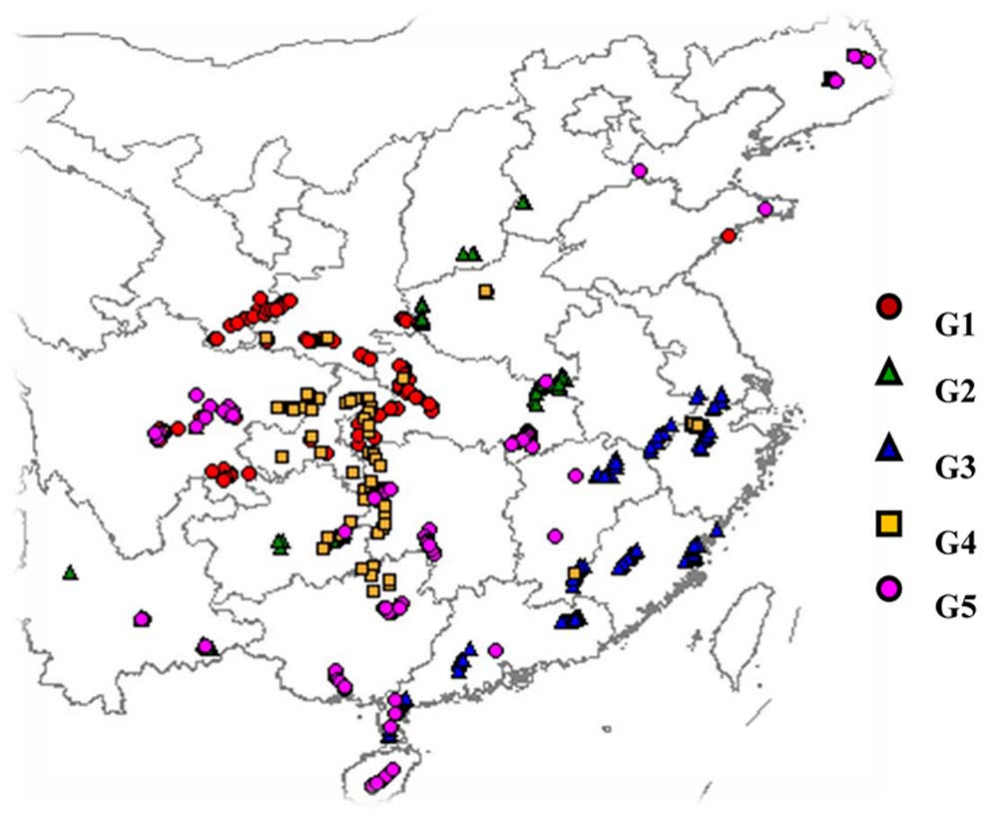
The original geographical distribution of 457 *M. sinensis* accessions showed on a partial Chinese map. The colorful objects represent various subpopulations generated by population structure analysis. The two accessions from USA are not shown in this figure.

According to the cutoff criterion for inferred ancestry estimate [32]–[33], majority of the accessions were clearly assigned to specific groups when the Q value was ≥0.6. The admixture between groups referred to 9 accessions when its Q value was <0.6 (Table S2). Accordingly, the 459 accessions were assigned into five subpopulations/groups, namely G1 (92 accessions), G2 (94 accessions), G3 (96 accessions), G4 (86 accessions), and G5 (91 accessions) (Fig. 2a). G1 accessions were mainly collected from the west to center of China. The genotypes of G2 were primarily collected from the north to center of China. G3 contained mostly accessions from the south to east. G4 accessions were mainly collected from the south to center. G5 had multiple origins covering a wide range of distribution areas. The genetic diversity was similar for the five groups, G1 (0.27), G2 (0.26), G3 (0.28), G4 (0.25) and G5 (0.26) (Table 1). The distance-based AMOVA analysis (Table 2) showed that both the within- and between-subpopulation genetic variations were significant, and the within-subpopulation (83%) was substantially greater than that of between-subpopulations (17%). Therefore, significant genetic differentiation occurred among the geographic regions in the panel of Chinese *M. sinensis* germplasm. According to the Nei’s genetic distance and pairwise *F_st_*, the genetic distance was the largest (0.22) between G1 and G4, and the smallest (0.12) between G3 and G5 (Table 3). In summary, high genetic diversity and substantial population differentiation occurred in *M*. *sinensis* germplasm collected in China, and the entire population could be divided into at least five variable subgroups.

**Table 2:** AMOVA analysis of genetic variation between *M. sinensis* subpopulations.

Source of variation		Degree of freedom	Square sum	Variance component	Percentage (%)
Among subpopulation		4	1262.28	3.27	17
Within subpopulation		454	7133.71	15.71	83
Total		458	8395.99	18.98	100
Fixation Index	Fst: 0.17				

**Table 3:** Genetic distance between *M. sinensis* subpopulations.

Group	G1	G2	G3	G4	G5
G1		0.0566	0.0537	0.0569	0.0524
G2	0.1901		0.0474	0.0518	0.0431
G3	0.1789	0.1635		0.0554	0.0354
G4	0.2166	0.1815	0.1906		0.0388
G5	0.1802	0.1515	0.1245	0.1432	

Nei’s minimum distance estimates list above the diagonal and pairwise *F_st_* lists below the diagonal.

### Classification and Geographic Distribution

Using the Neighbor-joining analysis, the 459 *M*. *sinensis* accessions were classified into five major branches (Fig. 1b). Result of this clustering was nearly the same as that of the structure analysis with a few exceptions (Fig. 1). All the G1 accessions except one were clustered into the red branch, and were mainly collected from Qinling Mountains.

In the green branch, 93 accessions were from the G2 subpopulation and contained nearly all those in the branch except for a single one from the G3 subpopulation. Most of the accessions were from the Yunnan-Guizhou plateau or He’nan province, and all the accessions from Shanxi and Hebei provinces were clustered into this branch.

Blue branch contained 94 accessions from the G3 and two from G5 subpopulation, respectively. Most of the accessions from the southeast China and four accessions from the southwest China were clustered into this branch.

Orange branch was consisted of 84 accessions from the G4 subpopulation and two accessions from the G2 subpopulation. In this branch, most accessions were from the central and south China, the northeast China, and the east China (Fig. 2).

Purple branch contained 91 accessions including 86 G5 accessions, two G4 accessions, and one accession from each of the G1, G2 and G3 subpopulation. Six accessions from Hainan province, two accessions from Shandong province, six of the nine Liaoning accessions, and two accessions from USA were clustered into this branch.

### Phenotypic Variation of Important Traits in the *M*. *sinensis* Germplasm

**Figure 3:**
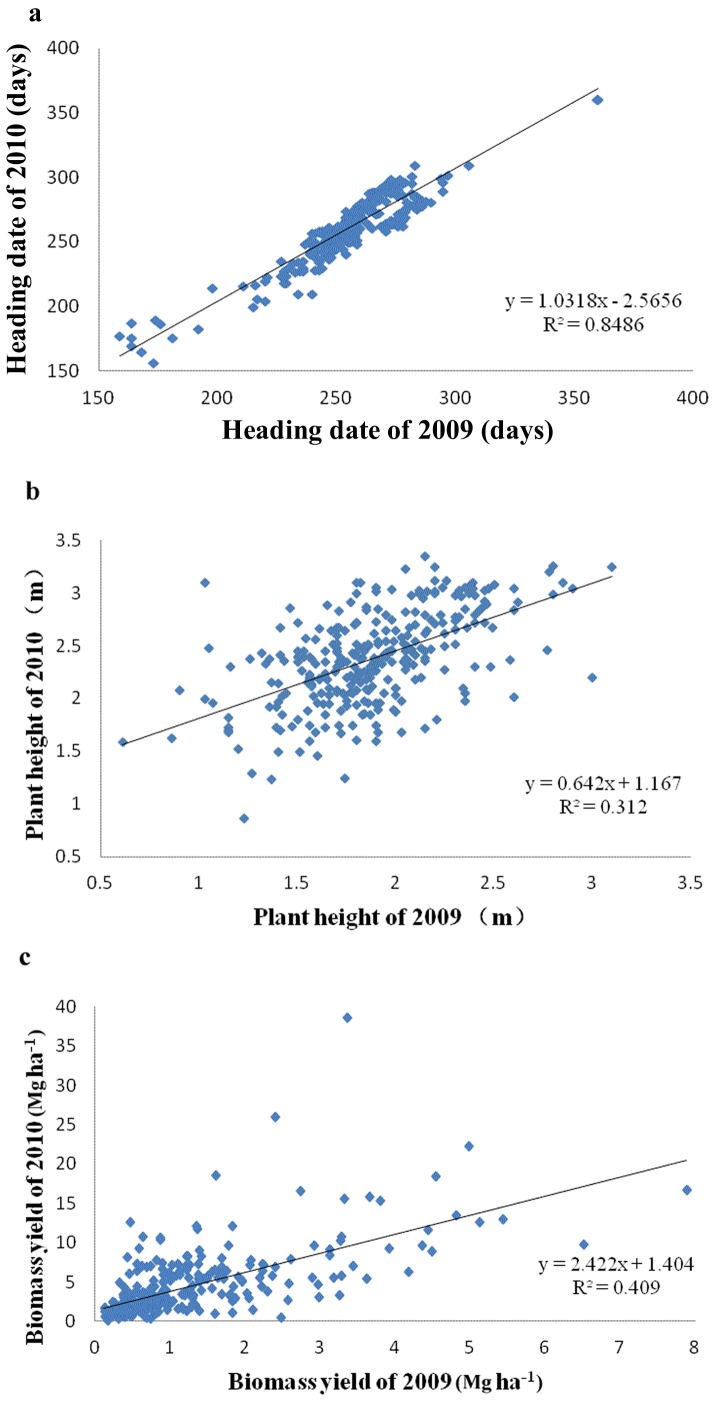
Phenotypic distribution of heading date, plant height and biomass yield in 300 *M*. *sinensis* accessions. a) heading date, b) plant height and c) biomass yield in the 1^st^ (2009) and 2^nd^ (2010) year after the Miscanthus field was established.

The phenotypic traits, heading date, plant height and biomass yield, were measured or recorded for 300 *M*. *sinensis* accessions over two years, the 1^st^ (2009) and 2^nd^ (2010) year after establishment of the *Miscanthus* field. Obvious phenotypic variations were observed for heading date (156–360 days), plant height (0.61–3.35 m), and biomass yield (0.12 to 38.49 Mg/ha) in the two years (Table S3). Regression analysis (Fig. 3) indicated that there were highly significant between-year correlations for the three observed traits, *r* = 0.9212, 0.6395 and 0.5586 for heading date, biomass yield and plant height, respectively. The between-year variation for heading date was small, indicating heading date of the 2^nd^ year was almost fully determined by the 1^st^ year with a determination coefficient *r^2^* = 0.849 (Fig. 3a). The between-year variation was much larger for biomass yield and plant height than heading date. Thus the performance in the 2^nd^ year could be greatly improved on the basis of the 1^st^ year with determination coefficient *r^2^* = 0.312 and 0.409 for plant height and biomass yield, respectively (Fig. 3b and 3c).

**Figure 4:**
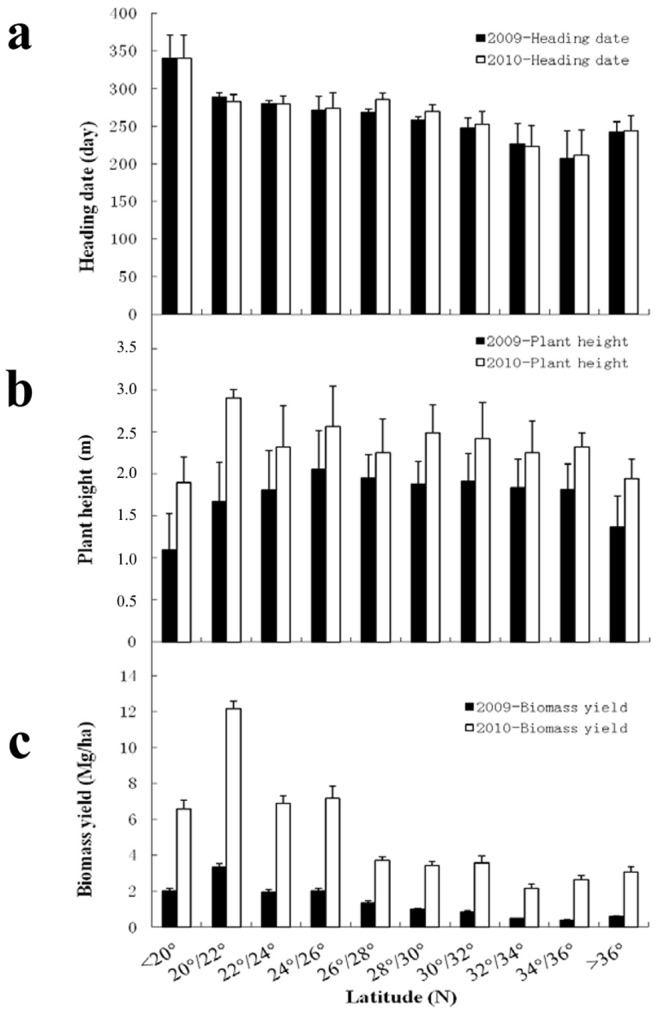
Phenotypic distribution against latitude (with interval of 2°) of the place of origin in Chinese *M*. *sinensis* germplasm. a) heading date, b) plant height and c) biomass yield in the 1^st^ (2009) and 2^nd^ (2010) year after the Miscanthus field was established.

The mean trait value of the *M*. *sinensis* accessions, collected from adjacent area within the interval of 2° latitude, was calculated and used to assess the relationship between latitude of the place of origin and the trait performance in Wuhan. The heading date showed similar pattern between 2009 and 2010, i.e., the heading date decreased with increase in latitude of the original site (Fig. 4a). There was no significant difference in heading date with 2° interval between the two years.

Plant height was positively correlated with biomass yield (*r* = 0.35). Therefore, plant height may be used as an indicator for selection of high biomass yield in *Miscanthus* breeding. The average plant height in 2010 was significant higher than that of 2009 across the regions from 18°50.076N to 40°32.196N (p<0.001). The plant height was relatively short for the accessions sampled outside the region of 20°-36°N, due to early heading (>36°N) and failure of transition from vegetative to reproductive growth (<20°N) respectively, indicating a close correlation between that the environmental adaptability and the original site (Fig. 4b). Significant difference in plant height was found with 2° interval between the two years (P<0.05).

High biomass yield is a base trait for the *Miscanthus* breeding and sustainable bioenergy industry. It is important to note there was a significant increase in biomass yield in the 2^nd^ (2010) than the 1^st^ year (2009) after the *Miscanthus* field establishment. An obvious trend of biomass decrease was observed in the two years with increase of the original latitude, except for the abnormal growth without successful reproductive stage of the accessions from Hainan (<20°N) (Fig. 4c). The accessions collected from 20–22°N had the highest averaged plant height and biomass yield, 2.91 m and 12.15 Mg ha^−1^, respectively (Fig. 4b, 4c). Significant difference in biomass yield was observed in every 2° interval between the two years (P<0.01).

### Marker-trait Association in the *M*. *sinensis* Germplasm

Based on the Q model, association analysis on the tested traits and the SSR markers were performed in the sample population of 300 *M*. *sinensis* accessions. A total of nine markers were found to be significantly associated with heading date and biomass yield (P<0.01). Among the associated markers, five were detected across the two years (Table 4). Four markers, DBM8-5, DBM18-1, DBM28-6 and DBM58-2 were associated with heading date, and one marker DBM55-5 was associated with biomass yield in the two years. Association of DBM29-4 and DBM28-5 with heading date, and that of DBM12-11 with biomass yield was only detected in 2010. The contribution of single associated markers to the phenotypic variation was relatively low, but the accumulated contribution should be high (Table 4).

**Table 4:** Alleles significantly associated with the observed traits in the *M*. *sinensis.*

Trait	SSR marker	Allele size (bp)	P value	Variance (%)	Year
Heading date	DBM18-1	122	9.99E-04	4.53	2009 and 2010
Heading date	DBM28-6	450	9.99E-04	2.64	2009 and 2010
Heading date	DBM58-2	273	9.99E-04	2.74	2009 and 2010
Heading date	DBM8-5	178	0.008	1.68	2009 and 2010
Heading date	DBM49-2	122	0.009	1.77	2009
Heading date	DBM29-4	102	9.99E-04	2.33	2010
Heading date	DBM28-5	446	0.006	1.93	2010
Biomass yield	DBM55-5	158	9.99E-04	2.11	2009 and 2010
Biomass yield	DBM12-11	192	0.007	2.56	2010

### Correlation and Regression Analyses on Biomass Yield and Other Parameters

To predict the relationship between biomass yield and geographic parameters of the original habitats, heading date and plant height, correlation and regression analyses were performed using the 300 *M*. *sinensis* accessions. Results showed that biomass yield was positively correlated with the heading date and plant height while negative correlations were observed with the original latitude and altitude in 2009 and 2010, respectively (Table 5). No significant linear relationships were detected between biomass yield and longitude (Table 5). Therefore, high biomass yield could depend largely on heading date and plant height, and was negatively impacted by the original latitude and altitude.

**Table 5:** Pearson correlation coefficients between geographical parameters of the original habitats and the observed traits in *M*. *sinensis*.

	Latitude	Longitude	Altitude	Heading date 2009	Plant height 2009	Biomass yield 2009	Heading date 2010	Plant height 2010	Biomass yield 2010
Latitude	/	/	/	-0.757**	NS	−0.507**	−0.691**	NS	−0.391**
Longitude		/	/	NS	NS	NS	NS	NS	NS
Altitude			/	−0.266**	−0.217**	−0.241**	−0.279**	−0.336**	−0.239**
Heading date 2009				/	NS	0.380**	0.921**	NS	0.304**
Plant height 2009					/	0.349**	NS	0.559**	0.230**
Biomass yield 2009						/	0.325**	0.379**	0.640**
Heading date 2010							/	NS	0.220**
Plant height 2010								/	0.468**
Biomass yield 2010									/

** Correlation is significant at the 0.01 level (two-tailed). NS means not significant.

Results of regression analysis showed that biomass yield (Y) increased linearly with plant height (x_1_) and decreased linearly with the original latitude (x_2_). Regression equations were obtained for 2009 and 2010, respectively, Y = 343.80+1.04x_1_−14.79x_2_ (*r* = 0.62, P<0.001, 2009) and Y = 520.71+4.31x_1_−39.24x_2_ (*r* = 0.59, P<0.001, 2010).

## Discussion

### Genetic Diversity and Differentiation of the Chinese *M*. *sinensis* Germplasm

The large difference of genetic diversity between the cultivated varieties and the wild germplasm could likely be attributed to domestication events that greatly reduced genetic diversity in popular varieties [34]–[36]. Evaluation of the genetic diversity in wild germplasm is crucial for efficient exploitation of the valuable alleles present in the wild resources, which has been demonstrated in previous studies for rice, maize and sweet sorghum [34], [37]–[42]. Transferable SSR markers have proved valuable for analyzing genetic diversity [30]. In the present study, we investigated the genetic diversity using 23 transferable SSR markers in a large panel of 459 *M*. *sinensis* accessions. The high diversity was observed (Table 2 and 3) and implied the great potential of its use in the genetic improvement of *Miscanthus*, including *M*. *sinensis* and *M.* × *giganteus*. Despite similar genetic diversity detected in the five subpopulations, unique alleles were observed only in two groups. These unique alleles specific to a couple of subpopulations and the detected rare alleles demonstrated a significant genetic differentiation in the *M*. *sinensis* germplasm that would be useful for broadening the genetic base of *Miscanthus* breeding pools.

### Molecular Classification, Population Structure and Geographic Distribution

The Chinese *M*. *sinensis* germplasm could be divided into 5 groups (Fig. 1a), which was highly consistent with the grouping result of structure analysis with few exceptions (Fig. 1b). Although no perfect correlation was observed between groups and geographical distributions of the *M*. *sinensis* germplasm, a strikingly nonrandom spatial pattern of genetic variation could be drawn. *M*. *sinensis* accessions (represented as blue triangle in Fig. 2) from southeast China were clustered into a big branch. Accessions collected in this area had greater genetic similarity and were distinct from other areas, indicating that significant gene flow occurred in this region.

Distributing in ellipse-like shape, red circle represents accessions grouped in G1 (Fig. 2). Except one accession, all other accessions were collected from the Qinling Mountain area. *M*. *sinensis* could provide food and shelter for vertebrates and birds, which might facilitate spreading of *M*. *sinensis* seeds in this area [43]–[44].

Most G4 accessions (presented in the yellow square in Fig. 1a and Fig. 2) were distributed across a wide zone which covers the central China and southern China. The zone is parallel with a major migratory path of migratory birds [45]. With awn, it is easy for *M*. *sinensis* seeds to stick on the birds and other vertebrates’ feather and thus be transported throughout the migratory path. Consequently the migratory birds might unconsciously contribute to the gene flow process of *M*. *sinensis* in this region.

All G2 accessions (represented by green triangle) were distributed in the Yun-Gui Plateau in southwest China and the areas around He’nan province in north-central China. The altitude of the Yun-Gui Plateau is high, with an average of 936 m in Guizhou and 1388 m in Yunnan of the collected accessions. Most accessions collected in this area were clustered together in this group (Fig. 2). He’nan province is an area with relatively high latitude, and almost all the accessions collected from this area were grouped into the same branch. The two branches then merged into a bigger branch. Noticing that the climate, especially temperature, at higher altitudes in the south might be similar to that at higher latitudes, we assumed that climate might be an important factor influencing the grouping pattern of *M*. *sinensis* in China.

In Fig. 2, accessions represented by purple circles were dispersedly distributed. Although the climate in northeast China is distinct from that of south China, as a large geographic distance separates the two areas, the genetic similarity of accessions from these two areas was very high. Accessions from the two areas were clustered together with accessions from south and central China into a big branch. The accessions in south and central China were grouped in four subpopulations, which might imply that there was a high genetic diversity of *M*. *sinensis* in south and central China. All these results indicated that Hainan island in south China and Liaoning province in northeast China could not possibly be the origin of *M*. *sinensis*, and it was not be too long ago that *M*. *sinensis* spread to these areas [46]–[48]. Otherwise, we should have gotten a specific branch that consists of accessions only from Hainan island or Liaoning province.

Furthermore, AMOVA analysis agreed with the clustering pattern, the NJ tree and the genetic distance measurements among the five subpopulations, implying that the 459 Chinese *M*. *sinensis* accessions were highly differentiated, possibly due to the ease in which the arista of *M*. *sinensis* seeds sticking to the bodies of animals, as well all the characteristics that *Miscanthus* being a wind pollinated genus [49]. The population structure of *M*. *sinensis* accessions could plausibly be attributed to seed-mediated and pollen-mediated gene flow from region to region. As is well known, among a large number of alleles, only few rare alleles could account for geographic differentiation in the process of speciation. Accordingly, genetic difference existed among subpopulations but most of the genetic variation was still maintained within subpopulations. Thus further studies should be conducted to better understand the mechanism of geographic differentiation of *M*. *sinensis* germplasm in China.

### Breeding for Biofuel

*M*. *sinensis* showed great potential to be feedstock for bioethanol [1]–[3]. However, obtaining maximum output from *M*. *sinensis* on a wider climate range and in various environments requires enlarged genetic variability [1]. The *M*. *sinensis* germplasm in this study harbored great genetic diversity, and showed significant genetic structural differentiation and significant phenotypic variation in heading date, plant height and biomass/feedstock yield. The information of the genetic structure and diversity would be useful for breeders to develop new *M*. *sinensis* cultivars and hybrids. In other words, the development of ideal cultivars and hybrids for industry purposes should greatly benefit from genetic knowledge of diverse germplasm collections at the beginning and breeding scheme design. Furthermore, researchers should understand genetic variation of quality and stress-tolerance traits of *M*. *sinensis*, including the component and degradation efficiency of lignocelluloses, and the tolerance to biotic and abiotic stresses. These traits are important in *Miscanthus* breeding for ideal biofuel feedstock.

### Prospects for Association Mapping

Identification of quantitative trait loci (QTLs) associated with plant height, panicle height, stem diameter and yield have been reported [20]–[21]. Transferable SSR markers from *B. distachyon* have been successfully used in the genetic diversity study on *M*. *sinensis* [30]. In this study, 23 transferable SSR markers from *B*. *distachyon* were used to estimate the genetic diversity and population structure of the Chinese *M*. *sinensis* germplasm. The neighbor-joining tree based on Nei’s [50] genetic distance revealed a division of 5 subpopulations/groups (Fig. 2b), demonstrating extensive genetic diversity (Table 1). Therefore, the molecular marker data along with phenotypic trait data were used to explore the marker-trait association. In the present study, *M*. *sinensis* showed an amazing degree of phenotypic polymorphism (Fig. 3 and 4). Significant associations of markers with heading date and biomass yield were found in the subsequent two years, based on 115 alleles from 23 SSR markers transferable from *B*. *distachyon* and the astonishing phenotypic variation. Since the proportion of significant associations was relatively high with the limited number of markers, we concluded that the association analysis was efficient for detecting true marker-trait relationships. Additionally, the results also demonstrated that the *M*. *sinensis* germplasm accessions could be exploited and applied in breeding programs in the future.

Research on *Miscanthus* as a biomass feedstock has greatly improved our understanding of biomass yield potential, agronomy and adaptation [51]. Since China cannot allow biomass energy production from the current fertile croplands, marginal lands may be used for biomass energy production. Therefore, multi-environment trial data and high genome-coverage SNP or SSR markers are needed to improve association of the marker loci or elite genes with the target traits.

## Materials and Methods

### Plant Materials and Phenotyping

Four hundred and fifty-nine *M*. *sinensis* accessions were used in this study, including 457 accessions collected from China and two accessions from USA (ornamental cultivars with fine slim leaf, Table S2 Table S2). The accessions were collected individually and covered the major distribution areas in China (Fig. 2). Over 10 *Miscanthus* rhizomes from an individual clone were sampled. The rhizome from each accession was split evenly and planted in a row with no less than 3 clonal replicates on Jan 18, 2009 (Wuhan, Hubei, China). Three phenotypic traits, including heading date, plant height and biomass yield, were recorded in 2009 and 2010. Heading date (HD) was recorded as the days after sprout to the date when the main stem start heading. *M*. *sinensis* is a short-day crop and its development is largely determined by the day-length. Due to this photoperiod requirement, days from sprout until reproductive growth were quite variable among the *M*. *sinensis* accessions collected from different geographical regions. Therefore, the accessions collected from Hainan (a short-day region) failed to heading when planted in Wuhan (a relatively long-day region). The heading date of several genotypes collected from Hainan was recorded as 360 days. Plant height (PH), was measured as the length from the ground to the tip of the panicle when the shoot was harvested in the following February. Biomass production (dry matter) per plant of each accession was assessed after air drying to constant weight. No specific permission was required for the field study, which was carried out in the experimental plot, a specific garden for cellulose energy plants. *M*. *sinensis* is not listed as the endangered or protected species in China.

### Genotyping with SSR Primers from *Brachypodium distachyon*

Genomic DNA was isolated from young leaves following a modified cetyltrimethyl ammonium bromide (CTAB) method [52]. The integrity and quality of the DNA was detected using 1.0% (w/v) agarose gel by comparing band intensity with concentration-known λDNA standard (Promega Corporation, Madison, USA).

A total of 23 SSR primer pairs developed from *B. distachyon* [30] were selected to genotype the 459 *M*. *sinensis* (Table S4). The polymerase chain reaction (PCR) reaction mixture contained 60 ng of *Miscanthus* genomic DNA with the following components in a 20 µL total volume: 1× PCR buffer with 1.8 mM MgCl_2_, 200 µM dNTP, 500 nM forward and reverse primers respective, and 1 unit of Taq DNA polymerase. The PCR amplifications were performed in an BIORAD-My Cycle Thermocyclers (Bio-Rad Laboratories, Inc., California, USA), applying a touchdown amplification protocol: start with pre-denaturation step at 95°C for 3 min; followed with ten touchdown cycles of 95°C for 30 s, 60°C (decreased 0.5°C per cycle) for 30 s, and 72°C for 30 s; then 30 cycles of denaturation at 95°C for 30 s, annealing at 50∼62°C (optimized annealing temperature for each pair of primer were listed in Table S4) for 30 s, and extension at 72°C for 30 s. The PCR amplifications were terminated with a final extension step at 72°C for 7 min. The PCR products were fractionated on a 6% denaturing acrylamide/bis gel with Liuyi-gel system (DYCZ-20E,Liuyi Instrument Factory,Beijing) under the condition of 80 W for 1.5 h. The gel with separated amplicons was visualized by silver staining. A 50 bp DNA ladder (Invitrogen, Carlsbad, CA) on the same gel was used as standard size marker to estimate the fragment sizes of the PCR products. The bands which represented amplicon size between 100–500 bp were recorded in a matrix of ‘1’ and ‘0’ to representing the presence or absence of amplification, respectively.

### Data Analysis

#### Cluster analysis

Several genetic parameters were estimated and cluster analysis was performed using the neighbour-joining method in PowerMarker version 3.25 [53], including the basic statistics of allele frequency of each loci, gene diversity and Chord distance [53]–[54]. The Chord distance matrix among accessions was used to draw the consensus neighbor-joining (NJ) tree. NJ tree was edited using FigTree v1.3.1 (http://tree.bio.ed.ac.uk/software/figtree/). Unique alleles were those present in one accession or one group of accessions but absent in other accessions or groups of accessions. Rare alleles were those with frequency of ≤5% in the investigated materials. Those alleles frequency >20% were classified as most frequent alleles.

#### Population structure

Our data was converted with the script AFLPdat [55]. The transformed data was coded with a top row setting 0 as the recessive allele, making all models available for dominant markers in STRUCTURE 2.3 [56]–[57]. The STRUCTURE program was implemented with Bayesian model-based clustering method to detect population structure and to assign individuals to subpopulations. The admixture model using correlated allele frequency was the preferred model that most accurately assigns individuals to closely related groups, and thus was used in the present study. The STRUCTURE program was run five times for each subpopulation value (k), ranging from 1 to 10, with a burn-in period of 10^4^ followed by 10^5^ iterations. Evanno’s delta K [58] was calculated to determine the optimum number of subpopulations. Out of the five runs for k = 5, the run with maximum likelihood value was chosen to assign accessions with the posterior membership coefficients (Q). A graphical bar plot which represented the posterior membership coefficients of each accession was then generated.

Analysis of molecular variance (AMOVA) was performed using Arlequin 3.11 [59] on the basis of pairwise distances, to assess population differentiation among different subpopulations. Furthermore, the genetic distances among these five subpopulations were calculated as Nei’s distance [60] and pairwise *F_st_*.

#### Correlation and regression analyses

Regression analysis of biomass yield on geographic parameters of the original habitats, heading date and plant height was carried out and the correlations were estimated as Pearson coefficient (r). All the analyses were performed using the statistical software SPSS13.0 for windows (SPSS Inc., Chicago, IL, USA).

#### Association analysis

Association analysis was conducted to reveal associations between the measured traits and the 115 marker alleles. A General linear model (GLM) test using posterior membership coefficients (population structure Q) as a cofactor was run in TASSEL 2.1 [61] following the user’s manual [62]. The P value showing significance of association between alleles and traits were estimated, as well as the R^2^ indicating the fixed marker effects.

## Supporting Information

Table S1 Geographical parameters and the group information for *M. sinensis* germplasm accessions.
(XLS)

Table S2 Primer sequences, optimal annealing temperature (AT), number of polymorphic bands (NB) and chromosome location on the *B. distachyon* for each primer pair.
(DOC)

Table S3 The phenotypic data, biomass yield, plant height and heading time for each *M*. *sinensis* accessions in 2009 and 2010.
(DOC)

Table S4 The genotypic data of 115 alleles in the *M*. *sinensis* accessions.
(DOC)
