# Supplementary material for: Genetic Diversity and Population Structure of Miscanthus sinensis Germplasm in China
Source: PLoS One. 2013 Oct 7;8(10):e75672. doi: 10.1371/journal.pone.0075672 (PMC3792140; doi:10.1371/journal.pone.0075672)
Supplement: Table S2 — Primer sequences, optimal annealing temperature (AT), number of polymorphic bands (NB) and chromosome location on the B. distachyon for each primer pair. (DOC) [file pone.0075672.s002.doc]

**Table S2** Geographical parameters and the group information for *M. sinensis* germplasm accessions

| Code^a^ | Origin^b^ | Latitude | Longitude | Ele^c^ | Gro^d^ | Q value |
| --- | --- | --- | --- | --- | --- | --- |
| ah1 | Xiuning county, Anhui | 29°29.243’N | 118°09.268’E | 270 | G3 | 0.985 |
| ah2 | Tunxi district, Anhui | 29°45.734’N | 118°21.494’E | 136 | G3 | 0.951 |
| ah3 | Xiuning county, Anhui | 29°28.142’N | 118°08.800’E | 425 | G3 | 0.950 |
| ah4 | Xiuning county, Anhui | 29°26.523’N | 118°09.225’E | 232 | G3 | 0.970 |
| ah5 | Xiuning county, Anhui | 29°38.607’N | 118°09.503’E | 176 | G3 | 0.978 |
| ah6 | Jixi county, Anhui | 30°04.703’N | 118°34.874’E | 193 | G3 | 0.987 |
| ah7 | Ningguo city, Anhui | 30°18.976’N | 118°46.413’E | 181 | G3 | 0.980 |
| ah8 | She county, Anhui | 29°57.446’N | 118°21.721’E | 145 | G3 | 0.965 |
| ah9 | Xiuning county, Anhui | 29°38.658’N | 118°11.224’E | 147 | G3 | 0.974 |
| ah10 | Jixi county, Anhui | 30°00.720’N | 118°32.715’E | 160 | G3 | 0.876 |
| cq1 | Fengdu county, Chongqing | 29°53.825’N | 107°41.725’E | 228 | G4 | 0.977 |
| cq2 | Fuling district, Chongqing | 29°50.146’N | 107°35.627’E | 201 | G1 | 0.945 |
| cq3 | Pengshui county, Chongqing | 29°22.523’N | 108°06.658’E | 282 | G1 | 0.918 |
| cq4 | Ba'nan district, Chongqing | 29°16.639’N | 106°44.508’E | 441 | G4 | 0.961 |
| cq5 | Fengjie county, Chongqing | 31°05.761’N | 109°28.729’E | 375 | G4 | 0.938 |
| cq6 | Fengjie county, Chongqing | 30°32.802’N | 109°21.495’E | 1523 | G4 | 0.980 |
| cq7 | Fengjie county, Chongqing | 30°48.022’N | 109°30.560’E | 371 | G4 | 0.990 |
| cq8 | Fengjie county, Chongqing | 30°40.829’N | 109°27.737’E | 1329 | G4 | 0.983 |
| cq9 | Liangping county, Chongqing | 30°39.393’N | 107°47.607’E | 525 | G4 | 0.946 |
| cq10 | Wulong county, Chongqing | 29°22.343’N | 107°56.600’E | 147 | G4 | 0.943 |
| cq11 | Youyang county, Chongqing | 28°47.838’N | 108°54.914’E | 406 | G4 | 0.980 |
| cq12 | Yunyang county, Chongqing | 31°07.774’N | 109° 01.246’E | 906 | G4 | 0.991 |
| cq13 | Yunyang county, Chongqing | 30°58.24’N | 108°44.613’E | 537 | G4 | 0.879 |
| cq14 | Yunyang county, Chongqing | 31°01.128’N | 108°51.994’E | 446 | G4 | 0.982 |
| fj1 | Fuding city, Zhejiang | 26°58.445’N | 120°11.661’E | 7 | G3 | 0.939 |
| fj2 | Fuzhou city, Fujian | 26°01.300’N | 119°11.839’E | 8 | G3 | 0.954 |
| fj3 | Fuzhou city, Fujian | 26°01.300’N | 119°11.839’E | 8 | G3 | 0.984 |
| fj4 | Fuzhou city, Fujian | 26°02.361’N | 119°29.941’E | 47 | G3 | 0.988 |
| fj5 | Fuzhou city, Fujian | 26°10.680’N | 119°33.341’E | 16 | G3 | 0.985 |
| fj6 | Lianjiang county, Fujian | 26°11.022’N | 119°32.383’E | 14 | G3 | 0.987 |
| fj7 | Luoyuan county, Fujian | 26°19.433’N | 119°29.942’E | 88 | G3 | 0.985 |
| fj8 | Luoyuan county, Fujian | 26°23.580’N | 119°28.717’E | 107 | G3 | 0.949 |
| fj9 | Luoyuan county, Fujian | 26°28.861’N | 119°31.130’E | 74 | G3 | 0.983 |
| fj10 | Ningde city, Fujian | 26°34.409’N | 119°36.538’E | 17 | G3 | 0.987 |
| fj11 | Ningde city, Fujian | 26°31.676’N | 119°37.946’E | 279 | G3 | 0.988 |
| fj12 | Sanming city, Fujian | 26°11.001’N | 117°32.834’E | 152 | G3 | 0.963 |
| fj13 | Sanming city, Fujian | 26°09.018’N | 117°30.187’E | 152 | G3 | 0.978 |
| fj14 | Sha county, Fujian | 26°18.918’N | 117°41.781’E | 133 | G3 | 0.981 |
| fj15 | Sha county, Fujian | 26°23.024’N | 117°46.091’E | 121 | G3 | 0.983 |
| fj16 | Yong'an city, Fujian | 26°02.917’N | 117°25.924’E | 164 | G3 | 0.985 |
| fj17 | Sanming city, Fujian | 26°05.940’N | 117°27.330’E | 162 | G3 | 0.961 |
| fj18 | Yong'an city, Fujian | 25°53.213’N | 117°15.909’E | 214 | G3 | 0.870 |
| fj19 | Yong'an city, Fujian | 25°56.897’N | 117°19.182’E | 191 | G3 | 0.971 |
| fj20 | Yong'an city, Fujian | 25°49.136’N | 117°11.460’E | 234 | G3 | 0.979 |
| fj21 | Yong'an city, Fujian | 25°45.878’N | 117°09.119’E | 261 | G3 | 0.984 |
| fj22 | Yong'an city, Fujian | 25°45.878’N | 117°09.119’E | 261 | G3 | 0.961 |
| gd1 | Guangzhou City, Hubei | 23°10.778’N | 113°22.725’E | 74 | G5 | 0.919 |
| gd2 | Guangzhou City, Hubei | 23°10.891’N | 113°22.691’E | 127 | G5 | 0.770 |
| gd3 | Guangzhou City, Hubei | 23°11.099’N | 113°22.677’E | 167 | G5 | 0.971 |
| gd4 | Guangzhou City, Hubei | 23°11.147’N | 113°22.795’E | 234 | G5 | 0.965 |
| gd5 | Guangzhou City, Hubei | 23°10.491’N | 113°22.780’E | 53 | G5 | 0.928 |
| gd6 | Longchuan county, Guangdong | 24°03.692’N | 115°23.850’E | 231 | G3 | 0.982 |
| gd7 | Huazhou city, Guangdong | 21°34.205’N | 110°29.512’E | 27 | G3 | 0.909 |
| gd8 | Huazhou city, Guangdong | 21°39.376’N | 110°36.210’E | 19 | G3 | 0.869 |
| gd9 | Leizhou city, Guangdong | 20°42.009’N | 110°03.270’E | 59 | G3 | 0.979 |
| gd10 | Leizhou city, Guangdong | 20°44.161’N | 110°03.815’E | 75 | G5 | 0.970 |
| gd11 | Leizhou city, Guangdong | 20°27.514’N | 110°01.590’E | 106 | G3 | 0.980 |
| gd12 | Leizhou city, Guangdong | 20°33.490’N | 110°04.990’E | 122 | G3 | 0.845 |
| gd13 | Lianjiang city, Guangdong | 21°38.131’N | 110°14.640’E | 37 | G5 | 0.973 |
| gd14 | Lianjiang city, Guangdong | 21°38.198’N | 110°14.741’E | 32 | G5 | 0.983 |
| gd15 | Mei county, Guangdong | 24°15.738’N | 115°57.890’E | 165 | G3 | 0.983 |
| gd16 | Wuhua county, Guangdong | 24°04.343’N | 115°37.535’E | 128 | G3 | 0.983 |
| gd17 | Wuhua county, Guangdong | 24°04.441’N | 115°29.176’E | 161 | G3 | 0.926 |
| gd18 | Wuhua county, Guangdong | 24°05.271’N | 115°32.246’E | 136 | G3 | 0.956 |
| gd19 | Xinxing county, Guangdong | 22°49.362’N | 112°13.779’E | 67 | G3 | 0.978 |
| gd20 | Xinxing county, Guangdong | 22°45.587’N | 112°13.665’E | 28 | G3 | 0.977 |
| gd21 | Xinxing county, Guangdong | 22°30.844’N | 112°11.776’E | 421 | G3 | 0.975 |
| gd22 | Xinxing county, Guangdong | 22°32.611’N | 112°12.161’E | 122 | G3 | 0.974 |
| gd23 | Xingning city, Guangdong | 24°07.051’N | 115°39.493’E | 159 | G3 | 0.986 |
| gd24 | Xingning city, Guangdong | 24°13.556’N | 115°55.911’E | 258 | G3 | 0.976 |
| gd25 | Xingning city, Guangdong | 24°12.731’N | 115°49.568’E | 222 | G3 | 0.940 |
| gd26 | Xingning city, Guangdong | 24°11.221’N | 115°49.455’E | 169 | G3 | 0.966 |
| gd27 | Yunfu city, Guangdong | 22°52.514’N | 112°16.819’E | 20 | G3 | 0.964 |
| gd28 | Yunfu city, Guangdong | 22°54.802’N | 112°20.218’E | 20 | G3 | 0.903 |
| gd29 | Zhanjiang city, Guangdong | 21°31.866’N | 110°25.323’E | 15 | G3 | 0.926 |
| gd30 | Zhanjiang city, Guangdong | 21°29.128’N | 110°22.207’E | 9 | G3 | 0.976 |
| gd31 | Zhanjiang city, Guangdong | 21°11.050’N | 110°17.517’E | 94 | G5 | 0.953 |
| gd32 | Zhanjiang city, Guangdong | 21°10.057’N | 110°17.639’E | 107 | G5 | 0.969 |
| gd33 | Zhanjiang city, Guangdong | 21°11.676’N | 110°14.693’E | 37 | G5 | 0.971 |
| gd34 | Zhaoqing city, Guangdong | 23°12.232’N | 112°34.566’E | 9 | G3 | 0.973 |
| gs1 | Cheng county, Gansu | 33°39.328’N | 105°32.350’E | 1352 | G1 | 0.976 |
| gs2 | Cheng county, Gansu | 33°41.550’N | 105°36.810’E | 1238 | G1 | 0.955 |
| gs3 | Wei county, Gansu | 33°58.152’N | 105°59.633’E | 1058 | G1 | 0.975 |
| gs4 | Wei county, Gansu | 33°45.460’N | 105°47.757’E | 1009 | G1 | 0.963 |
| gs5 | Wei county, Gansu | 33°49.896’N | 106°11.285’E | 826 | G1 | 0.972 |
| gs6 | Kang county, Gansu | 33°30.149’N | 105°21.374’E | 1116 | G1 | 0.989 |
| gs7 | Liangdang county, Gansu | 33°55.268’N | 106°15.362’E | 1316 | G1 | 0.986 |
| gs8 | Wen county, Gansu | 33°03.083’N | 104°39.089’E | 1801 | G1 | 0.982 |
| gs9 | Wen county, Gansu | 33°03.179’N | 104°40.050’E | 1962 | G1 | 0.955 |
| gs10 | Wudu district, Gansu | 33°27.329’N | 105°08.243’E | 1807 | G1 | 0.981 |
| gx1 | Guilin city, Guangxi | 25° 12.679’N | 110° 02.895’E | 152 | G4 | 0.848 |
| gx2 | lin'gui county, Guangxi | 25° 21.113’N | 110° 06.298’E | 145 | G4 | 0.975 |
| gx3 | Rong'an county, Guangxi | 25° 00.968’N | 109° 35.114’E | 499 | G4 | 0.946 |
| gx4 | Sanjiang county, Guangxi | 25° 46.364’N | 109° 35.539’E | 162 | G4 | 0.973 |
| gx5 | Sanjiang county, Guangxi | 25° 28.399’N | 109° 29.658’E | 219 | G4 | 0.977 |
| gx6 | Sanjiang county, Guangxi | 25° 42.468’N | 109° 15.192’E | 165 | G4 | 0.970 |
| gx7 | Lipu county, Guangxi | 24°41.231’N | 110°28.313’E | 176 | G5 | 0.990 |
| gx8 | Lipu county, Guangxi | 24°32.181’N | 110°23.740’E | 155 | G5 | 0.981 |
| gx9 | Lipu county, Guangxi | 24°35.083’N | 110°25.784’E | 155 | G4 | 0.948 |
| gx10 | Lipu county, Guangxi | 24°38.967’N | 110°25.799’E | 167 | G5 | 0.964 |
| gx11 | Lipu county, Guangxi | 24°25.343’N | 110°12.797’E | 255 | G5 | 0.989 |
| gx12 | Jinxiu county, Guangxi | 24°23.680’N | 110°07.844’E | 263 | G5 | 0.967 |
| gx13 | Jinxiu county, Guangxi | 24°20.245’N | 110°04.598’E | 218 | G5 | 0.988 |
| gx14 | Liuzhou city, Guangxi | 24°32.325’N | 109°51.216’E | 138 | G5 | 0.955 |
| gx15 | Liuzhou city, Guangxi | 24°24.286’N | 109°58.826’E | 164 | G5 | 0.934 |
| gx16 | Liuzhou city, Guangxi | 24°30.576’N | 109°56.977’E | 157 | G5 | 0.974 |
| gx17 | Nanning city, Guangxi | 22°18.315’N | 108°24.274’E | 49 | G5 | 0.983 |
| gx18 | Nanning city, Guangxi | 22°23.642’N | 108°22.859’E | 137 | G5 | 0.951 |
| gx19 | Nanning city, Guangxi | 22°29.801’N | 108°24.013’E | 128 | G5 | 0.982 |
| gx20 | Qinzhou city, Guangxi | 22°07.516’N | 108°37.000’E | 38 | G5 | 0.970 |
| gx21 | Qinzhou city, Guangxi | 22°13.850’N | 108°25.247’E | 53 | G5 | 0.968 |
| gx22 | Qinzhou city, Guangxi | 22°11.630’N | 108°31.450’E | 25 | G5 | 0.979 |
| gx23 | Qinzhou city, Guangxi | 21°56.444’N | 108°38.726’E | 3 | G5 | 0.984 |
| gx24 | Qinzhou city, Guangxi | 21°59.491’N | 108°39.910’E | 16 | G5 | 0.971 |
| gx25 | Qinzhou city, Guangxi | 22°01.883’N | 108°37.833’E | 36 | G5 | 0.975 |
| gz1 | Huaxi district, Guizhou | 26°23.970’N | 106°39.848’E | 1091 | G2 | 0.976 |
| gz2 | Huaxi district, Guizhou | 26°24.215’N | 106°39.982’E | 1111 | G2 | 0.933 |
| gz3 | Huaxi district, Guizhou | 26°23.971’N | 106°39.905’E | 1096 | G2 | 0.888 |
| gz4 | Huaxi district, Guizhou | 26°23.721’N | 106°39.749’E | 1091 | G2 | 0.965 |
| gz5 | Jinyang district, Guizhou | 26°38.333’N | 106°37.152’E | 1300 | G2 | 0.979 |
| gz6 | Jinyang district, Guizhou | 26°38.466’N | 106°36.423’E | 1303 | G2 | 0.973 |
| gz7 | Jinyang district, Guizhou | 26°38.768’N | 106°36.417’E | 1301 | G2 | 0.943 |
| gz8 | Jinyang district, Guizhou | 26°39.355’N | 106°36.829’E | 1297 | G2 | 0.982 |
| gz9 | Jinyang district, Guizhou | 26°38.919’N | 106°36.873’E | 1284 | G2 | 0.981 |
| gz10 | Wudang district, Guizhou | 26°36.890’N | 106°44.904’E | 1095 | G2 | 0.983 |
| gz11 | Wudang district, Guizhou | 26°37.118’N | 106°45.477’E | 1102 | G2 | 0.988 |
| gz12 | Wudang district, Guizhou | 26°37.047’N | 106°45.781’E | 1066 | G2 | 0.979 |
| gz13 | Wudang district, Guizhou | 26°37.233’N | 106°45.087’E | 1133 | G2 | 0.768 |
| gz14 | Wudang district, Guizhou | 26°37.026’N | 106°45.052’E | 1073 | G2 | 0.967 |
| gz15 | Jianhe county, Guizhou | 26°48.094’N | 108°34.012’E | 583 | G2 | 0.973 |
| gz16 | Jianhe county, Guizhou | 26°42.378’N | 108°23.672’E | 672 | G2 | 0.961 |
| gz17 | Jianhe county, Guizhou | 26° 47.886’N | 108° 33.882’E | 596 | G4 | 0.955 |
| gz18 | Jianhe county, Guizhou | 26°45.137’N | 108°29.884’E | 512 | G2 | 0.983 |
| gz19 | Kaili city, Guizhou | 26°35.950’N | 108°01.206’E | 752 | G2 | 0.964 |
| gz20 | Leishan county, Guizhou | 26° 19.306’N | 108° 03.504’E | 984 | G4 | 0.982 |
| gz21 | Sansui county, Guizhou | 26°55.706’N | 108°38.982’E | 620 | G2 | 0.651 |
| gz22 | Sansui county, Guizhou | 26°53.675’N | 108°37.272’E | 632 | G2 | 0.971 |
| gz23 | Sansui county, Guizhou | 26°56.977’N | 108°40.007’E | 631 | G2 | 0.978 |
| gz24 | Sansui county, Guizhou | 26°50.311’N | 108°35.531’E | 608 | G2 | 0.949 |
| gz25 | Songtao county, Guizhou | 28° 12.037’N | 109° 16.806’E | 722 | G4 | 0.964 |
| gz26 | Songtao county, Guizhou | 27° 56.95’N | 109° 16.907’E | 626 | G4 | 0.978 |
| gz27 | Taijiang county, Guizhou | 26°39.131’N | 108°13.791’E | 691 | G2 | 0.971 |
| gz28 | Taijiang county, Guizhou | 26°40.359’N | 108°08.864’E | 786 | G2 | 0.985 |
| gz29 | Taijiang county, Guizhou | 26° 40.783’N | 108° 10.578’E | 625 | G4 | 0.971 |
| gz30 | Taijiang county, Guizhou | 26° 40.783’N | 108° 10.578’E | 625 | G4 | 0.971 |
| gz31 | Taijiang county, Guizhou | 26°38.905’N | 108°16.855’E | 666 | G2 | 0.985 |
| gz32 | Taijiang county, Guizhou | 26°38.035’N | 108°06.989’E | 832 | G2 | 0.913 |
| gz33 | Yuping county, Guizhou | 27° 13.134’N | 108° 52.143’E | 384 | G4 | 0.987 |
| hb1 | Badong county, Hubei | 30°47.859’N | 110°15.746’E | 1758 | G1 | 0.978 |
| hb2 | Chibi city, Hubei | 29°44.020’N | 113°56.110’E | 45 | G5 | 0.975 |
| hb3 | Chibi city, Hubei | 29°46.106’N | 114°02.606’E | 71 | G5 | 0.969 |
| hb4 | Chibi city, Hubei | 29°47.972’N | 114°07.676’E | 42 | G5 | 0.964 |
| hb5 | Chibi city, Hubei | 29°50.543’N | 114°13.084’E | 50 | G5 | 0.896 |
| hb6 | Chibi city, Hubei | 29°43.105’N | 113°50.837’E | 46 | G5 | 0.864 |
| hb7 | Enshi city, Hubei | 30° 26.48’N | 109° 19.891’E | 1727 | G4 | 0.989 |
| hb8 | Enshi city, Hubei | 30° 16.01’N | 109° 27.608’E | 430 | G4 | 0.912 |
| hb9 | Fang county, Hubei | 31°51.735’N | 110°31.177’E | 1495 | G1 | 0.957 |
| hb10 | Fang county, Hubei | 32°04.235’N | 110°31.710’E | 559 | G1 | 0.967 |
| hb11 | Fang county, Hubei | 32°04.908’N | 110°29.885’E | 600 | G1 | 0.944 |
| hb12 | Fang county, Hubei | 32°06.912’N | 110°27.580’E | 588 | G1 | 0.982 |
| hb13 | Hong'an county, Hubei | 31°05.036’N | 114°33.907’E | 69 | G2 | 0.976 |
| hb14 | Hong'an county, Hubei | 31°28.678’N | 114°43.574’E | 86 | G2 | 0.969 |
| hb15 | Hong'an county, Hubei | 31°27.144’N | 114°38.601’E | 76 | G2 | 0.979 |
| hb16 | Hong'an county, Hubei | 30°58.654’N | 114°38.578’E | 43 | G2 | 0.956 |
| hb17 | Hong'an county, Hubei | 31°19.789’N | 114°38.334’E | 69 | G2 | 0.931 |
| hb18 | Jianshi county, Hubei | 30°35.033’N | 109°42.297’E | 583 | G1 | 0.952 |
| hb19 | Jianshi county, Hubei | 30°47.754’N | 110°03.322’E | 1728 | G1 | 0.961 |
| hb20 | Lichuan city, Hubei | 30°19.619’N | 109°04.419’E | 1037 | G1 | 0.972 |
| hb21 | Macheng county, Hubei | 31°32.505’N | 115°08.947’E | 163 | G2 | 0.989 |
| hb22 | Macheng county, Hubei | 31°35.960’N | 115°09.517’E | 231 | G2 | 0.988 |
| hb23 | Shenlongjia district, Hubei | 31°28.204’N | 110°23.278’E | 1258 | G1 | 0.941 |
| hb24 | Shenlongjia district, Hubei | 31°36.080’N | 110°23.907’E | 1526 | G1 | 0.985 |
| hb25 | Shenlongjia district, Hubei | 31°29.613’N | 110°22.023’E | 1499 | G1 | 0.985 |
| hb26 | Shenlongjia district, Hubei | 31°29.831’N | 110°22.259’E | 1608 | G1 | 0.918 |
| hb27 | Shenlongjia district, Hubei | 31°33.161’N | 110°20.846’E | 1678 | G1 | 0.895 |
| hb28 | Shenlongjia district, Hubei | 31°44.313’N | 110°28.518’E | 1786 | G4 | 0.886 |
| hb29 | Shenlongjia district, Hubei | 31°45.487’N | 110°32.291’E | 1480 | G1 | 0.983 |
| hb30 | Shenlongjia district, Hubei | 31°45.403’N | 110°32.570’E | 1400 | G1 | 0.985 |
| hb31 | Shenlongjia district, Hubei | 31°45.257’N | 110°37.493’E | 1017 | G1 | 0.982 |
| hb32 | Tongshan county, Hubei | 29°36.257’N | 114°29.130’E | null | G4 | 0.917 |
| hb33 | Tongshan county, Hubei | 29°36.29’N | 114°29.180’E | null | G4 | 0.964 |
| hb34 | Tongshan county, Hubei | 29°36.34’N | 114°29.150’E | null | G4 | 0.923 |
| hb35 | Tongshan county, Hubei | 29°36.54’N | 114°29.230’E | null | G4 | 0.937 |
| hb36 | Tongshan county, Hubei | 29°36.35’N | 114°29.450’E | null | G4 | 0.972 |
| hb37 | Tongshan county, Hubei | 29°36.61’N | 114°29.370’E | null | G4 | 0.983 |
| hb38 | Jiangxia district, Hubei | 29°36.33’N | 114°29.090’E | null | G5 | 0.979 |
| hb39 | Xianfeng county, Hubei | 29°39.421’N | 109°07.168’E | 770 | G1 | 0.976 |
| hb40 | Xianfeng county, Hubei | 29°59.417’N | 109°03.432’E | 684 | G1 | 0.955 |
| hb41 | Xianning city, Hubei | 29°50.257’N | 114°19.450’E | null | G4 | 0.978 |
| hb42 | Xianning city, Hubei | 29°50.800’N | 114°19.760’E | null | G4 | 0.96 |
| hb43 | Xianning city, Hubei | 29°52.257’N | 114°19.120’E | null | G4 | 0.984 |
| hb44 | Xianning city, Hubei | 30°07.476’N | 114°21.126’E | 40 | G5 | 0.723 |
| hb45 | Xianning city, Hubei | 30°03.380’N | 114°21.282’E | 42 | G5 | 0.956 |
| hb46 | Xianning city, Hubei | 29°56.814’N | 114°21.763’E | 41 | G5 | 0.968 |
| hb47 | Xianning city, Hubei | 29°53.855’N | 114°17.498’E | 41 | G5 | 0.975 |
| hb48 | Xingshan county, Hubei | 31°11.968’N | 110°55.393’E | 514 | G1 | 0.982 |
| hb49 | Shenlongjia district, Hubei | 31°20.228’N | 110°33.667’E | 596 | G1 | 0.976 |
| hb50 | Xingshan county, Hubei | 31°21.667’N | 110°37.295’E | 371 | G1 | 0.992 |
| hb51 | Xingshan county, Hubei | 31°08.815’N | 110°50.500’E | 171 | G1 | 0.989 |
| hb52 | Xuan'en county, Hubei | 29°53.773’N | 109°33.660’E | 723 | G1 | 0.975 |
| hb53 | Xuan'en county, Hubei | 30° 03.993’N | 109° 26.014’E | 692 | G4 | 0.969 |
| hb54 | Yichang city, Hubei | 30°51.832’N | 111°21.653’E | 145 | G1 | 0.931 |
| hb55 | Yichang city, Hubei | 30°45.904’N | 111°20.426’E | 119 | G1 | 0.966 |
| hb56 | Yichang city, Hubei | 30°56.374’N | 111°17.194’E | 220 | G1 | 0.988 |
| hb57 | Zhushan county, Hubei | 32°10.333’N | 110°21.159’E | 576 | G1 | 0.962 |
| heb1 | Neiqiu county, Hebei | 37°20.214’N | 114°16.784’E | 345 | G2 | 0.978 |
| heb2 | Neiqiu county, Hebei | 37°20.419’N | 114°15.981’E | 468 | G2 | 0.891 |
| heb3 | Neiqiu county, Hebei | 37°20.710’N | 114°14.878’E | 458 | G2 | 0.948 |
| heb4 | Neiqiu county, Hebei | 37°19.941’N | 114°11.576’E | 455 | G2 | 0.96 |
| hen1 | Xixia county, Henan | 33°30.670’N | 111°04.333’E | 475 | G2 | 0.981 |
| hen2 | Lushi county, Henan | 34°07.769’N | 111°02.637’E | 757 | G2 | 0.98 |
| hen3 | Lushi county, Henan | 33°38.173’N | 111°02.256’E | 707 | G2 | 0.983 |
| hen4 | Lushi county, Henan | 33°34.349’N | 111°03.372’E | 541 | G2 | 0.976 |
| hen5 | Lushi county, Henan | 33°35.549’N | 111°02.004’E | 582 | G2 | 0.979 |
| hen6 | Lushi county, Henan | 34°06.067’N | 111°03.383’E | 726 | G2 | 0.944 |
| hen7 | Lushi county, Henan | 33°44.782’N | 111°02.632’E | 796 | G2 | 0.980 |
| hen8 | Lushi county, Henan | 33°41.583’N | 111°04.886’E | 844 | G2 | 0.986 |
| hen9 | Shangcheng county, Henan | 31°34.763’N | 115°20.218’E | 179 | G2 | 0.988 |
| hen10 | Shangcheng county, Henan | 31°32.863’N | 115°18.734’E | 155 | G2 | 0.980 |
| hen11 | Shangcheng county, Henan | 31°47.131’N | 115°26.521’E | 111 | G2 | 0.954 |
| hen12 | Shangcheng county, Henan | 31°53.949’N | 115°25.260’E | 91 | G2 | 0.961 |
| hen13 | Shangcheng county, Henan | 31°52.680’N | 115°24.702’E | 84 | G2 | 0.982 |
| hen14 | Shangcheng county, Henan | 31°45.393’N | 115°28.393’E | 137 | G2 | 0.984 |
| hen15 | Shangcheng county, Henan | 31°44.851’N | 115°29.294’E | 205 | G2 | 0.981 |
| hen16 | Shangcheng county, Henan | 31°44.494’N | 115°29.376’E | 252 | G2 | 0.971 |
| hen17 | Shangcheng county, Henan | 31°44.177’N | 115°29.291’E | 420 | G2 | 0.958 |
| hen18 | Shangcheng county, Henan | 31°44.305’N | 115°29.636’E | 476 | G2 | 0.987 |
| hen19 | Shangcheng county, Henan | 31°45.261’N | 115°32.880’E | 330 | G2 | 0.982 |
| hen20 | Shangcheng county, Henan | 31°45.526’N | 115°32.105’E | 295 | G2 | 0.972 |
| hen21 | Shangcheng county, Henan | 31°45.817’N | 115°31.886’E | 193 | G2 | 0.92 |
| hen22 | Shangcheng county, Henan | 31°47.474’N | 115°25.440’E | 113 | G2 | 0.965 |
| hen23 | Shangcheng county, Henan | 31°47.480’N | 115°25.429’E | 104 | G2 | 0.976 |
| hen24 | Shangcheng county, Henan | 31°30.882’N | 115°12.814’E | 289 | G2 | 0.937 |
| hen25 | Shangcheng county, Henan | 31°30.650’N | 115°17.389’E | 156 | G2 | 0.988 |
| hen26 | Shangcheng county, Henan | 31°30.557’N | 115°17.922’E | 161 | G2 | 0.981 |
| hen27 | Shangcheng county, Henan | 31°28.899’N | 115°19.000’E | 230 | G2 | 0.974 |
| hen28 | Xin county, Henan | 31°42.059’N | 114°55.873’E | 122 | G2 | 0.97 |
| hen29 | Xin county, Henan | 31°42.079’N | 114°52.447’E | 103 | G5 | 0.483 |
| hen30 | Xin county, Henan | 31°41.741’N | 115°01.266’E | 110 | G2 | 0.951 |
| hen31 | Xin county, Henan | 31°34.528’N | 115°04.352’E | 115 | G2 | 0.984 |
| hen32 | Xin county, Henan | 31°34.405’N | 114°50.020’E | 178 | G2 | 0.974 |
| hen33 | Xin county, Henan | 31°36.810’N | 114°51.558’E | 96 | G2 | 0.988 |
| hen34 | Dengfeng city, Henan | 34°30.270’N | 113°02.495’E | 1291 | G4 | 0.969 |
| hen35 | Dengfeng city, Henan | 34°30.270’N | 113°02.495’E | 1291 | G1 | 0.988 |
| hen36 | Dengfeng city, Henan | 34°30.341’N | 113°02.462’E | 1371 | G2 | 0.983 |
| hen37 | Dengfeng city, Henan | 34°30.352’N | 113°02.461’E | 1382 | G2 | 0.876 |
| hen38 | Dengfeng city, Henan | 34°30.388’N | 113°02.475’E | 1389 | G2 | 0.972 |
| hen39 | Dengfeng city, Henan | 34°30.426’N | 113°02.507’E | 1397 | G2 | 0.976 |
| hen40 | Dengfeng city, Henan | 34°30.440’N | 113°02.511’E | 1385 | G2 | 0.957 |
| hen41 | Dengfeng city, Henan | 34°30.468’N | 113°02.515’E | 1391 | G2 | 0.899 |
| hen42 | Dengfeng city, Henan | 34°30.529’N | 113°02.517’E | 1413 | G2 | 0.988 |
| hen43 | Dengfeng city, Henan | 34°30.531’N | 113°02.489’E | 1428 | G2 | 0.974 |
| hen44 | Dengfeng city, Henan | 34°30.660’N | 113°02.444’E | 1488 | G2 | 0.986 |
| hen45 | Dengfeng city, Henan | 34°30.270’N | 113°02.495’E | 1291 | G2 | 0.883 |
| hn1 | Qiongzhong county, Hainan | 19°02.486’N | 109°46.605’E | 307 | G5 | 0.979 |
| hn2 | Qiongzhong county, Hainan | 19°09.529’N | 109°55.512’E | 256 | G5 | 0.914 |
| hn3 | Tunchang county, Hainan | 19°23.708’N | 110°09.104’E | 103 | G5 | 0.958 |
| hn4 | Wuzhishan city, Hainan | 18°50.076’N | 109°30.565’E | 781 | G5 | 0.961 |
| hn5 | Wuzhishan city, Hainan | 18°54.335’N | 109°30.827’E | 309 | G5 | 0.973 |
| hn6 | Wuzhishan city, Hainan | 19°00.588’N | 109°38.781’E | 343 | G5 | 0.977 |
| hun1 | Dong'an county, Hu'nan | 26°24.988’N | 111°16.873’E | 181 | G5 | 0.927 |
| hun2 | Dong'an county, Hu'nan | 26°19.091’N | 111°25.630’E | 124 | G5 | 0.985 |
| hun3 | Dong'an county, Hu'nan | 26°14.502’N | 111°27.224’E | 119 | G5 | 0.967 |
| hun4 | Dong'an county, Hu'nan | 26°22.372’N | 111°22.407’E | 137 | G5 | 0.941 |
| hun5 | Dong'an county, Hu'nan | 26°28.526’N | 111°15.210’E | 402 | G5 | 0.978 |
| hun6 | Fenghuang county, Hu'nan | 28°03.960’N | 109°35.328’E | 365 | G5 | 0.975 |
| hun7 | Fenghuang county, Hu'nan | 27°59.583’N | 109°35.490’E | 365 | G5 | 0.963 |
| hun8 | Hongjiang city, Hu'nan | 27° 07.888’N | 109° 57.076’E | 215 | G4 | 0.959 |
| hun9 | Huaheng county, Hu'nan | 28° 31.171’N | 109° 29.145’E | 487 | G4 | 0.93 |
| hun10 | Huaihua city, Hu'nan | 27° 20.085’N | 109° 54.718’E | 224 | G4 | 0.969 |
| hun11 | Huitong county, Hu'nan | 27° 01.069’N | 109° 50.316’E | 374 | G4 | 0.974 |
| hun12 | Huitong county, Hu'nan | 26° 50.786’N | 109° 41.689’E | 296 | G4 | 0.987 |
| hun13 | Jishou city, Hu'nan | 28° 20.860’N | 109° 32.943’E | 745 | G4 | 0.980 |
| hun14 | Jishou city, Hu'nan | 28°16.275’N | 109°46.983’E | 177 | G5 | 0.963 |
| hun15 | Jishou city, Hu'nan | 28°13.605’N | 109°49.337’E | 178 | G5 | 0.973 |
| hun16 | Jishou city, Hu'nan | 28°14.195’N | 109°40.677’E | 199 | G4 | 0.750 |
| hun17 | Longshan county, Hubei | 29° 24.029’N | 109° 28.120’E | 582 | G4 | 0.981 |
| hun18 | Longshan county, Hubei | 29°20.555’N | 109° 36.259’E | 467 | G4 | 0.981 |
| hun19 | Longshan county, Hubei | 29° 09.949’N | 109° 37.807’E | 563 | G4 | 0.754 |
| hun20 | Luxi county, Hu'nan | 28°14.015’N | 109°52.523’E | 157 | G5 | 0.979 |
| hun21 | Luxi county, Hu'nan | 28°14.846’N | 109°56.328’E | 151 | G5 | 0.975 |
| hun22 | Luxi county, Hu'nan | 28°14.144’N | 109°58.178’E | 137 | G5 | 0.970 |
| hun23 | Luxi county, Hu'nan | 28°15.104’N | 110°06.928’E | 124 | G5 | 0.927 |
| hun24 | Mayang county, Hu'nan | 27° 50.869’N | 109° 45.914’E | 196 | G4 | 0.985 |
| hun25 | Shaoyang city, Hu'nan | 26°47.818’N | 111°12.415’E | 311 | G5 | 0.978 |
| hun26 | Shaoyang city, Hu'nan | 27°00.140’N | 111°17.003’E | 247 | G5 | 0.983 |
| hun27 | Xinhua city, Hu'nan | 27° 40.763’N | 109° 55.085’E | 253 | G4 | 0.944 |
| hun28 | Xingning county, Hu'nan | 26°44.934’N | 111°09.037’E | 339 | G5 | 0.982 |
| hun29 | Xingning county, Hu'nan | 26°39.919’N | 111°11.809’E | 321 | G5 | 0.933 |
| hun30 | Xingning county, Hu'nan | 26°33.619’N | 111°13.337’E | 376 | G5 | 0.937 |
| hun31 | Yongshun county, Hu'nan | 28° 59.129’N | 109° 49.954’E | 365 | G4 | 0.989 |
| js1 | Suzhou city, Jiangsu | 31°06.862’N | 120°23.734’E | 21 | G3 | 0.973 |
| js2 | Suzhou city, Jiangsu | 31°02.827’N | 120°23.270’E | 6 | G3 | 0.967 |
| js3 | Suzhou city, Jiangsu | 31°03.890’N | 120°23.697’E | 11 | G3 | 0.979 |
| js4 | Suzhou city, Jiangsu | 31°18.836’N | 120°23.996’E | 12 | G3 | 0.764 |
| js5 | Suzhou city, Jiangsu | 31°14.861’N | 120°24.113’E | 14 | G3 | 0.987 |
| js6 | Yixing city, Jiangsu | 31°20.744’N | 119°41.059’E | 22 | G3 | 0.908 |
| js7 | Yixing city, Jiangsu | 31°10.465’N | 119°35.131’E | 76 | G3 | 0.944 |
| jx1 | Huchang county, Jiangxi | 25°35.120’N | 115°47.845’E | 169 | G3 | 0.975 |
| jx2 | Huchang county, Jiangxi | 25°26.670’N | 115°46.841’E | 198 | G3 | 0.542 |
| jx3 | Huchang county, Jiangxi | 25°14.616’N | 115°44.613’E | 228 | G3 | 0.932 |
| jx4 | Huchang county, Jiangxi | 25°29.620’N | 115°45.947’E | 182 | G3 | 0.979 |
| jx5 | Huchang county, Jiangxi | 25°24.428’N | 115°46.777’E | 207 | G3 | 0.953 |
| jx6 | Jingdezhen city, Jiangxi | 29°15.672’N | 117°06.379’E | 51 | G3 | 0.943 |
| jx7 | Jingdezhen city, Jiangxi | 29°12.272’N | 117°03.999’E | 30 | G3 | 0.913 |
| jx8 | Leping city, Jiangxi | 29°00.579’N | 117°08.036’E | 25 | G3 | 0.695 |
| jx9 | Leping city, Jiangxi | 29°06.069’N | 117°04.831’E | 86 | G3 | 0.952 |
| jx10 | Nanchang city, Jiangxi | 28°41.072’N | 115°51.670’E | null | G5 | 0.803 |
| jx11 | Ruichang city, Jiangxi | 25°35.120’N | 115°47.850’E | null | G4 | 0.986 |
| jx12 | Ruichang city, Jiangxi | 25°35.120’N | 115°47.850’E | null | G4 | 0.882 |
| jx13 | Ruijin city, Jiangxi | 25°52.441’N | 116°07.149’E | 258 | G3 | 0.949 |
| jx14 | Ruijin city, Jiangxi | 25°48.099’N | 115°58.449’E | 199 | G3 | 0.969 |
| jx15 | Ruijin city, Jiangxi | 25°51.456’N | 116°00.167’E | 193 | G3 | 0.907 |
| jx16 | Ruijin city, Jiangxi | 25°43.946’N | 115°53.358’E | 192 | G3 | 0.914 |
| jx17 | Ruijin city, Jiangxi | 25°39.687’N | 115°48.985’E | 180 | G3 | 0.982 |
| jx18 | Taihe county, Jiangxi | 26°46.083’N | 115°10.673’E | 118 | G5 | 0.961 |
| jx19 | Wangnian county, Jiangxi | 28°43.695’N | 116°54.241’E | 30 | G3 | 0.963 |
| jx20 | Wangnian county, Jiangxi | 28°43.581’N | 116°59.235’E | 58 | G3 | 0.979 |
| jx21 | Yugan county, Jiangxi | 28°43.874’N | 116°26.474’E | 32 | G3 | 0.969 |
| jx22 | Yugan county, Jiangxi | 28°42.778’N | 116°49.945’E | 21 | G3 | 0.923 |
| jx23 | Yugan county, Jiangxi | 28°44.516’N | 116°32.370’E | 18 | G3 | 0.972 |
| jx24 | Yugan county, Jiangxi | 28°42.561’N | 116°45.135’E | 16 | G3 | 0.723 |
| ln1 | Benxi city, Liaoning | 41°14.344’N | 123°41.169’E | 219 | G5 | 0.458 |
| ln2 | Benxi city, Liaoning | 41°13.918’N | 123°45.412’E | 282 | G4 | 0.987 |
| ln3 | Benxi city, Liaoning | 41°11.179’N | 123°49.110’E | 223 | G5 | 0.584 |
| ln4 | Benxi city, Liaoning | 41°09.679’N | 123°54.986’E | 360 | G5 | 0.936 |
| ln5 | Benxi city, Liaoning | 41°11.163’N | 123°50.752’E | 246 | G4 | 0.984 |
| ln6 | Qingyuan county, Liaoning | 41°59.756’N | 124°29.864’E | 157 | G4 | 0.587 |
| ln7 | Qingyuan county, Liaoning | 41°59.768’N | 124°29.859’E | 157 | G5 | 0.923 |
| ln8 | Qingyuan county, Liaoning | 41°54.836’N | 124°39.938’E | 385 | G5 | 0.551 |
| ln9 | Qingyuan county, Liaoning | 41°51.147’N | 124°56.308’E | 556 | G5 | 0.871 |
| sc1 | Chengdu city, Sichuan | 30°30.821’N | 104°21.574’E | 560 | G1 | 0.931 |
| sc2 | Chengdu city, Sichuan | 30°33.585’N | 104°12.336’E | 492 | G1 | 0.886 |
| sc3 | Chengdu city, Sichuan | 30°33.324’N | 104°18.273’E | 680 | G5 | 0.931 |
| sc4 | Chengdu city, Sichuan | 30°33.381’N | 104°18.276’E | 690 | G4 | 0.945 |
| sc5 | Da county, Sichuan | 31°12.790’N | 107° 34.744’E | 324 | G4 | 0.989 |
| sc6 | Da county, Sichuan | 31° 08.722’N | 107° 34.010’E | 309 | G4 | 0.986 |
| sc7 | Da county, Sichuan | 31° 13.968’N | 107° 31.404’E | 280 | G4 | 0.978 |
| sc8 | Daying county, Sichuan | 30°36.565’N | 105°12.378’E | 327 | G5 | 0.936 |
| sc9 | Daying county, Sichuan | 30°34.596’N | 105°14.573’E | 397 | G1 | 0.897 |
| sc10 | Dazhu county, Sichuan | 30° 49.434’N | 107° 18.416’E | 361 | G4 | 0.962 |
| sc11 | Jiang'an county, Sichuan | 28°46.573’N | 105°04.955’E | 354 | G1 | 0.985 |
| sc12 | Jiang'an county, Sichuan | 28°41.072’N | 105°08.090’E | 395 | G1 | 0.978 |
| sc13 | Jintang county, Sichuan | 30°53.299’N | 104°29.320’E | 576 | G5 | 0.925 |
| sc14 | Kaijiang county, Sichuan | 31° 04.732’N | 107° 52.588’E | 383 | G4 | 0.976 |
| sc15 | Kaijiang county, Sichuan | 30° 46.341’N | 107° 40.128’E | 500 | G4 | 0.962 |
| sc16 | Lushan county, Sichuan | 30°01.550’N | 102°53.856’E | 639 | G5 | 0.972 |
| sc17 | Lushan county, Sichuan | 30°03.884’N | 102°53.731’E | 636 | G5 | 0.955 |
| sc18 | Lushan county, Sichuan | 30°09.931’N | 102°53.713’E | 775 | G1 | 0.986 |
| sc19 | Mingshan county, Sichuan | 30°04.349’N | 103°02.688’E | 1105 | G1 | 0.96 |
| sc20 | Mingshan county, Sichuan | 30°04.947’N | 103°05.044’E | 672 | G1 | 0.949 |
| sc21 | Mingshan county, Sichuan | 30°04.636’N | 103°05.374’E | 648 | G1 | 0.969 |
| sc22 | Mingshan county, Sichuan | 30°04.462’N | 103°02.580’E | 1182 | G4 | 0.775 |
| sc23 | Nanxi county, Sichuan | 28°56.534’N | 104°54.637’E | 416 | G1 | 0.943 |
| sc24 | Nanxi county, Sichuan | 28°55.214’N | 104°54.863’E | 338 | G1 | 0.945 |
| sc25 | Peng'an county, Sichuan | 30° 49.719’N | 106° 35.777’E | 497 | G4 | 0.982 |
| sc26 | Pujiang county, Sichuan | 30°13.083’N | 103°28.878’E | 569 | G1 | 0.966 |
| sc27 | Qu county, Sichuan | 30°47.468’N | 107° 01.208’E | 328 | G4 | 0.978 |
| sc28 | Qu county, Sichuan | 30°46.738’N | 107° 04.609’E | 734 | G4 | 0.977 |
| sc29 | Santai county, Sichuan | 30°44.119’N | 105°10.640’E | 347 | G5 | 0.961 |
| sc30 | Santai county, Sichuan | 30°55.641’N | 105°03.662’E | 407 | G5 | 0.914 |
| sc31 | Shifang city, Sichuan | 30°16.548’N | 104°02.048’E | 716 | G5 | 0.869 |
| sc32 | Shifang city, Sichuan | 30°16.548’N | 104°02.048’E | 716 | G3 | 0.774 |
| sc33 | Shifang city, Sichuan | 31°15.836’N | 104°02.452’E | 693 | G5 | 0.775 |
| sc34 | Tianquan county, Sichuan | 29°55.492’N | 102°50.518’E | 718 | G5 | 0.923 |
| sc35 | Tianquan county, Sichuan | 30°01.375’N | 102°47.294’E | 718 | G5 | 0.967 |
| sc36 | Ya'an city, Sichuan | 29°57.687’N | 102°56.605’E | 645 | G1 | 0.979 |
| sc37 | Ya'an city, Sichuan | 29°52.709’N | 102°55.568’E | 882 | G1 | 0.98 |
| sc38 | Yibing city, Sichuan | 28°45.384’N | 105°41.575’E | 332 | G1 | 0.966 |
| sc39 | Yibing city, Sichuan | 28°50.751’N | 104°32.168’E | 398 | G1 | 0.981 |
| sc40 | Yingjing county, Sichuan | 29°49.415’N | 102°50.380’E | 745 | G1 | 0.974 |
| sc41 | Yingjing county, Sichuan | 29°50.541’N | 102°53.955’E | 1035 | G1 | 0.957 |
| sc42 | Changning county, Sichuan | 28°39.202’N | 104°56.959’E | 263 | G1 | 0.954 |
| sc43 | Changning county, Sichuan | 28°35.201’N | 104°52.759’E | 318 | G1 | 0.973 |
| sc44 | Zhongjiang county, Sichuan | 30°37.746’N | 105°03.713’E | 370 | G5 | 0.946 |
| sc45 | Zhongjiang county, Sichuan | 30°44.953’N | 104°56.253’E | 361 | G5 | 0.971 |
| sd1 | Yantai City, Shandong | 37°10.360’N | 121°44.050’E | 19.9 | G5 | 0.908 |
| sd2 | Qingdao City, Shandong | 36°16.159’N | 120°35.008’E | 199 | G1 | 0.437 |
| sd3 | Taishan City, Shandong | 36°14.000’N | 117°05’E | 550 | G5 | 0.742 |
| shx1 | Pingli county, Shaanxi | 32°26.310’N | 109°17.023’E | 440 | G1 | 0.973 |
| shx2 | Pingli county, Shaanxi | 32°31.201’N | 109°08.925’E | 476 | G1 | 0.977 |
| shx3 | Pingli county, Shaanxi | 32°21.038’N | 109°28.528’E | 546 | G1 | 0.944 |
| shx4 | Shiquan county, Shaanxi | 33°02.076’N | 108°09.034’E | 472 | G1 | 0.856 |
| shx5 | Shiquan county, Shaanxi | 33°01.938’N | 108°08.847’E | 476 | G1 | 0.986 |
| shx6 | Shiquan county, Shaanxi | 33°00.439’N | 108°08.616’E | 486 | G4 | 0.929 |
| shx7 | Shiquan county, Shaanxi | 33°00.439’N | 108°08.616’E | 486 | G1 | 0.987 |
| shx8 | Shiquan county, Shaanxi | 33°02.155’N | 108°13.270’E | 422 | G1 | 0.988 |
| shx9 | Shiquan county, Shaanxi | 32°57.560’N | 108°19.403’E | 403 | G1 | 0.983 |
| shx10 | Feng county, Shaanxi | 33°59.110’N | 106°39.505’E | 1033 | G1 | 0.987 |
| shx11 | Feng county, Shaanxi | 34°08.258’N | 106°45.272’E | 1190 | G1 | 0.971 |
| shx12 | Feng county, Shaanxi | 34°12.029’N | 106°51.387’E | 1353 | G1 | 0.983 |
| shx13 | Feng county, Shaanxi | 34°01.813’N | 106°39.506’E | 1042 | G1 | 0.979 |
| shx14 | Feng county, Shaanxi | 33°55.256’N | 106°25.370’E | 968 | G1 | 0.987 |
| shx15 | Feng county, Shaanxi | 33°55.660’N | 106°33.408’E | 973 | G1 | 0.979 |
| shx16 | Feng county, Shaanxi | 34°14.698’N | 106°55.988’E | 1467 | G1 | 0.990 |
| shx17 | Weibing district, Shaanxi | 34°15.751’N | 106°58.484’E | 1093 | G1 | 0.990 |
| shx18 | Weibing district, Shaanxi | 34°17.160’N | 106°03.105’E | 748 | G1 | 0.987 |
| shx19 | Chenggu county, Shaanxi | 33°02.995’N | 107°25.899’E | 583 | G1 | 0.985 |
| shx20 | Ningqiang county, Shaanxi | 33°01.479’N | 106°15.52’E | 750 | G4 | 0.980 |
| shx21 | Ningqiang county, Shaanxi | 33°01.479’N | 106°15.52’E | 750 | G4 | 0.937 |
| shx22 | Ningqiang county, Shaanxi | 33°00.161’N | 106°15.583’E | 755 | G1 | 0.901 |
| shx23 | Ningqiang county, Shaanxi | 33°01.479’N | 106°15.520’E | 750 | G1 | 0.969 |
| shx24 | Ningqiang county, Shaanxi | 32°58.878’N | 106°15.460’E | 797 | G1 | 0.985 |
| shx25 | Ningqiang county, Shaanxi | 32°57.850’N | 106°14.433’E | 1031 | G1 | 0.988 |
| shx26 | Xixiang county, Shaanxi | 33°00.927’N | 107°58.819’E | 665 | G1 | 0.954 |
| shx27 | Xixiang county, Shaanxi | 33°01.182’N | 108°04.978’E | 546 | G1 | 0.985 |
| shx28 | Xixiang county, Shaanxi | 32°58.200’N | 107°40.300’E | 458 | G1 | 0.987 |
| shx29 | Xixiang county, Shaanxi | 32°57.666’N | 107°35.558’E | 480 | G1 | 0.975 |
| shx30 | Xixiang county, Shaanxi | 32°57.404’N | 107°32.943’E | 489 | G1 | 0.986 |
| shx31 | Feng county, Shaanxi | 33°38.633’N | 110°28.134’E | 771 | G1 | 0.968 |
| shx32 | Feng county, Shaanxi | 33°37.930’N | 110°30.322’E | 667 | G1 | 0.987 |
| shx33 | Feng county, Shaanxi | 33°36.120’N | 110°36.794’E | 501 | G1 | 0.979 |
| shx34 | Shangnan county, Shaanxi | 33°31.379’N | 110°55.015’E | 513 | G1 | 0.991 |
| sx1 | Qinshui county, Shanxi | 35°43.358’N | 112°41.267’E | 941 | G2 | 0.958 |
| sx2 | Qinshui county, Shanxi | 35°42.326’N | 112°36.852’E | 783 | G2 | 0.956 |
| sx3 | Qinshui county, Shanxi | 35°43.318’N | 112°19.486’E | 724 | G2 | 0.965 |
| usa1 | USA | 40°32.196’N | 105°06.222’W | 1557 | G3 | 0.627 |
| usa2 | USA | 40°32.196’N | 105°06.222’W | 1557 | G3 | 0.515 |
| yn1 | Dali city, Yunnan | 25°40.743’N | 100°10.027’E | 2109 | G5 | 0.959 |
| yn2 | Eshan county, Yunnan | 24°11.183’N | 102°23.118’E | 1552 | G5 | 0.982 |
| yn3 | Eshan county, Yunnan | 24°10.221’N | 102°24.018’E | 1581 | G2 | 0.800 |
| yn4 | Eshan county, Yunnan | 24°11.510’N | 102°22.938’E | 1556 | G2 | 0.642 |
| yn5 | Eshan county, Yunnan | 24°11.654’N | 102°22.646’E | 1566 | G5 | 0.933 |
| yn6 | Wenshan county, Yunnan | 23°15.212’N | 104°27.173’E | 1389 | G2 | 0.945 |
| yn7 | Wenshan county, Yunnan | 23°19.498’N | 104°20.161’E | 1364 | G3 | 0.634 |
| yn8 | Wenshan county, Yunnan | 23°15.745’N | 104°25.271’E | 1408 | G2 | 0.953 |
| yn9 | Wenshan county, Yunnan | 23°15.174’N | 104°26.132’E | 1411 | G5 | 0.869 |
| yn10 | Wenshan county, Yunnan | 23°20.617’N | 104°17.983’E | 1289 | G2 | 0.985 |
| yn11 | Wenshan county, Yunnan | 23°19.446’N | 104°20.039’E | 1299 | G2 | 0.982 |
| yn12 | Wenshan county, Yunnan | 23°19.019’N | 104°21.794’E | 1389 | G2 | 0.960 |
| yn13 | Wenshan county, Yunnan | 23°18.160’N | 104°23.422’E | 1419 | G2 | 0.917 |
| yn14 | Wenshan county, Yunnan | 23°16.093’N | 104°25.107’E | 1409 | G2 | 0.954 |
| yn15 | Xichou county, Yunnan | 23°15.338’N | 104°33.846’E | 1138 | G2 | 0.903 |
| zj1 | Fuyang city, Zhejiang | 29°55.801’N | 119°52.975’E | 7 | G2 | 0.884 |
| zj2 | Fuyang city, Zhejiang | 29°53.506’N | 119°50.503’E | 20 | G2 | 0.974 |
| zj3 | Fuyang city, Zhejiang | 29°58.189’N | 119°54.843’E | 16 | G3 | 0.981 |
| zj4 | Fuyang city, Zhejiang | 30°06.202’N | 119°56.405’E | 10 | G3 | 0.916 |
| zj5 | Hangzhou city, Zhejiang | 30°08.894’N | 119°59.672’E | 70 | G3 | 0.978 |
| zj6 | Huzhou city, Zhejiang | 30°49.444’N | 120°04.461’E | 3 | G3 | 0.932 |
| zj7 | Huzhou city, Zhejiang | 30°52.463’N | 120°08.694’E | 10 | G3 | 0.934 |
| zj8 | Jiande city, Zhejiang | 29°35.575’N | 119°43.880’E | 252 | G3 | 0.757 |
| zj9 | Lin'an City, Zhejiang | 30°14.730’N | 119°41.470’E | 63 | G3 | 0.857 |
| zj10 | Lin'an City, Zhejiang | 30° 18.100’N | 119°30.400’E | 219 | G3 | 0.984 |
| zj11 | Lin'an City, Zhejiang | 30°12.670’N | 119°35.150’E | 101 | G4 | 0.945 |
| zj12 | Lin'an City, Zhejiang | 30°21.790’N | 119°28.390’E | 425 | G4 | 0.964 |
| zj13 | Lin'an City, Zhejiang | 30°16.440’N | 119°43.350’E | 67 | G4 | 0.976 |
| zj14 | Lin'an City, Zhejiang | 30° 18.300’N | 119°34.130’E | 206 | G4 | 0.987 |
| zj15 | Lin'an City, Zhejiang | 30° 18.270’N | 119°34.100’E | 197 | G4 | 0.990 |
| zj16 | Lin'an City, Zhejiang | 30°13.710’N | 119°47.640’E | 63 | G4 | 0.979 |
| zj17 | Lin'an City, Zhejiang | 30°12.090’N | 119°42.650’E | 51 | G4 | 0.490 |
| zj18 | Lin'an City, Zhejiang | 30°09.240’N | 119°42.200’E | 183 | G4 | 0.928 |
| zj19 | Lin'an City, Zhejiang | 30°14.170’N | 119°28.09’E | 200 | G4 | 0.956 |
| zj20 | Lin'an City, Zhejiang | 30°14.170’N | 119°28.09’E | 200 | G4 | 0.975 |
| zj21 | Lin'an City, Zhejiang | 30°17.900’N | 119°27.04’E | 275 | G4 | 0.935 |
| zj22 | Lin'an City, Zhejiang | 30°18.350’N | 119°29.54’E | 261 | G4 | 0.926 |
| zj23 | Pujiang county, Zhejiang | 29°33.636’N | 119°45.622’E | 651 | G4 | 0.973 |
| zj24 | Tonglu county, Zhejiang | 29°41.767’N | 119°40.129’E | 35 | G4 | 0.957 |
| zj25 | Tonglu county, Zhejiang | 29°48.164’N | 119°42.681’E | 29 | G3 | 0.977 |
| zj26 | Tonglu county, Zhejiang | 29°51.496’N | 119°46.399’E | 22 | G3 | 0.972 |

^a^ Accessions coded as abbreviation of provinces or states

^b^ Collection sites, including 2 accessions from USA

^c^ Elevation of collecting sites, ‘null’ means no record

^d^ Subpopulations
