# Supplementary material for: Genetic Diversity and Population Structure of Miscanthus sinensis Germplasm in China
Source: PLoS One. 2013 Oct 7;8(10):e75672. doi: 10.1371/journal.pone.0075672 (PMC3792140; doi:10.1371/journal.pone.0075672)
Supplement: Table S3 — The phenotypic data, biomass yield, plant height and heading time for each M. sinensis accessions in 2009 and 2010. (DOC) [file pone.0075672.s003.doc]

**Table S3** The phenotypic data, biomass yield, plant height and heading date for each *M*. *sinensis* accessions in 2009 and 2010.

|  |  | Biomass yield (Mg ha^-1^) | | Plant height (m) | | Heading date (d) | |
| --- | --- | --- | --- | --- | --- | --- | --- |
|  | Code | 2009 | 2010 | 2009 | 2010 | 2009 | 2010 |
| PMS-5 | hb52 | 0.33 | 0.98 | 1.60 | 1.96 | 251 | 248 |
| PMS-8 | hb19 | 0.74 | 0.34 | 1.86 | 2.08 | 257 | 250 |
| PMS-9 | hb18 | 3.25 | 3.32 | 1.82 | 2.38 | 259 | 251 |
| PMS-11 | hb20 | 2.10 | 1.39 | 2.08 | 2.45 | 254 | 255 |
| PMS-13 | hb40 | 1.85 | 2.76 | 1.95 | 2.43 | 259 | 274 |
| PMS-14 | hb39 | 1.79 | 3.23 | 2.49 | 2.68 | 259 | 277 |
| PMS-18 | cq3 | 0.90 | 4.98 | 2.45 | 2.93 | 265 | 286 |
| PMS-22 | cq2 | 1.28 | 1.77 | 2.45 | 2.72 | 261 | 278 |
| PMS-23 | cq1 | 1.83 | 1.03 | 2.06 | 2.60 | 264 | 274 |
| PMS-38 | sc9 | 1.25 | 3.55 | 2.06 | 2.37 | 264 | 278 |
| PMS-41 | sc38 | 0.35 | 0.68 | 1.81 | 2.25 | 268 | 272 |
| PMS-44 | sc12 | 0.80 | 1.03 | 1.77 | 2.13 | 265 | 275 |
| PMS-52 | sc39 | 0.68 | 1.40 | 1.87 | 2.20 | 267 | 271 |
| PMS-54 | sc20 | 1.37 | 5.43 | 2.15 | 3.35 | 261 | 279 |
| PMS-58 | sc18 | 0.68 | 6.81 | 2.18 | 2.75 | 263 | 264 |
| PMS-66 | sc2 | 1.82 | 2.60 | 2.85 | 3.10 | 260 | 260 |
| PMS-67 | sc4 | 3.62 | 5.41 | 2.77 | 2.46 | 265 | 284 |
| PMS-69 | sc1 | 2.36 | 5.72 | 2.45 | 3.03 | 260 | 270 |
| PMS-80 | hb55 | 1.39 | 1.33 | 1.74 | 2.65 | 257 | 270 |
| PMS-81 | hb54 | 1.71 | 5.41 | 1.77 | 2.06 | 254 | 264 |
| PMS-82 | hb56 | 1.84 | 2.80 | 2.05 | 2.45 | 254 | 261 |
| PMS-83 | hb48 | 0.68 | 2.03 | 1.86 | 2.08 | 254 | 269 |
| PMS-84 | hb51 | 0.58 | 2.30 | 1.53 | 2.40 | 256 | 262 |
| PMS-85 | hb50 | 0.81 | 2.11 | 2.32 | 2.78 | 249 | 243 |
| PMS-86 | hb49 | 0.47 | 3.32 | 1.90 | 2.34 | 227 | 223 |
| PMS-88 | hb25 | 0.22 | 1.11 | 1.57 | 2.19 | 247 | 239 |
| PMS-89 | hb26 | 0.68 | 2.67 | 1.91 | 2.52 | 243 | 234 |
| PMS-90 | hb27 | 0.31 | 1.50 | 1.64 | 2.35 | 234 | 234 |
| PMS-91 | hb24 | 0.52 | 1.92 | 1.90 | 2.32 | 211 | 215 |
| PMS-92 | hb28 | 0.31 | 0.29 | 1.81 | 2.21 | 174 | 189 |
| PMS-93 | hb29 | 0.50 | 1.44 | 1.70 | 2.68 | 216 | 216 |
| PMS-94 | hb30 | 0.71 | 1.76 | 1.71 | 2.83 | 221 | 222 |
| PMS-95 | hb31 | 0.55 | 1.50 | 1.50 | 1.80 | 245 | 243 |
| PMS-99 | hb9 | 0.25 | 1.01 | 1.66 | 2.10 | 245 | 258 |
| PMS-100 | hb10 | 0.47 | 0.75 | 1.74 | 2.04 | 246 | 247 |
| PMS-102 | hb12 | 0.45 | 2.03 | 1.72 | 1.89 | 229 | 223 |
| PMS-103 | hb57 | 0.66 | 0.57 | 1.03 | 2.00 | 253 | 244 |
| PMS-107 | shx3 | 0.68 | 0.50 | 1.66 | 1.95 | 249 | 246 |
| PMS-108 | shx1 | 0.59 | 2.12 | 2.34 | 2.10 | 250 | 253 |
| PMS-110 | shx2 | 0.51 | 3.53 | 1.73 | 2.34 | 246 | 239 |
| PMS-111 | shx9 | 0.57 | 3.56 | 1.86 | 2.25 | 247 | 236 |
| PMS-112 | shx8 | 0.36 | 1.09 | 1.69 | 2.26 | 233 | 226 |
| PMS-113 | shx4 | 0.28 | 1.21 | 1.70 | 2.53 | 246 | 244 |
| PMS-114 | shx5 | 0.40 | 3.19 | 2.13 | 2.48 | 239 | 242 |
| PMS-115 | shx7 | 0.60 | 1.97 | 1.89 | 2.35 | 245 | 242 |
| PMS-116 | shx27 | 1.15 | 1.84 | 2.62 | 2.92 | 240 | 247 |
| PMS-117 | shx26 | 0.79 | 4.45 | 2.39 | 3.10 | 253 | 250 |
| PMS-118 | shx28 | 1.36 | 11.65 | 2.38 | 3.08 | 241 | 249 |
| PMS-119 | shx29 | 0.89 | 3.42 | 2.35 | 3.06 | 245 | 255 |
| PMS-120 | shx30 | 0.23 | 1.73 | 2.23 | 2.80 | 250 | 244 |
| PMS-121 | shx19 | 0.56 | 7.19 | 1.56 | 2.20 | 247 | 249 |
| PMS-122 | shx23 | 0.56 | 4.43 | 1.94 | 2.42 | 245 | 248 |
| PMS-123 | shx22 | 0.88 | 2.36 | 1.40 | 1.97 | 244 | 258 |
| PMS-124 | shx24 | 0.79 | 1.20 | 1.91 | 2.55 | 198 | 214 |
| PMS-125 | shx25 | 0.27 | 0.97 | 1.86 | 1.96 | 176 | 186 |
| PMS-126 | gs8 | 0.13 | 1.72 | 1.56 | 2.18 | 173 | 156 |
| PMS-127 | gs9 | 0.25 | 2.27 | 1.37 | 2.16 | 229 | 218 |
| PMS-131 | gs6 | 0.47 | 2.28 | 1.91 | 2.37 | 228 | 218 |
| PMS-132 | gs1 | 0.13 | 0.57 | 1.65 | 2.18 | 220 | 204 |
| PMS-133 | gs2 | 0.21 | 0.52 | 1.60 | 1.46 | 215 | 199 |
| PMS-134 | gs4 | 0.39 | 0.54 | 1.90 | 1.96 | 159 | 177 |
| PMS-136 | gs5 | 0.26 | 2.14 | 1.55 | 2.16 | 228 | 222 |
| PMS-138 | shx14 | 0.30 | 1.19 | 1.83 | 2.39 | 181 | 175 |
| PMS-139 | shx15 | 0.47 | 1.08 | 1.58 | 2.03 | 217 | 205 |
| PMS-140 | shx10 | 0.22 | 0.53 | 1.90 | 2.27 | 192 | 182 |
| PMS-152 | hen1 | 0.26 | 0.89 | 2.21 | 1.80 | 234 | 209 |
| PMS-153 | hen4 | 0.17 | 0.52 | 1.80 | 2.08 | 240 | 209 |
| PMS-154 | hen5 | 0.42 | 2.92 | 2.13 | 2.20 | 164 | 175 |
| PMS-156 | hen8 | 0.22 | 0.78 | 1.56 | 1.75 | 220 | 219 |
| PMS-157 | hen7 | 0.17 | 0.12 | 1.47 | 1.74 | 236 | 228 |
| PMS-158 | hen6 | 0.18 | 1.03 | 1.78 | 2.43 | 237 | 248 |
| PMS-159 | hen2 | 0.55 | 6.36 | 2.08 | 2.47 | 164 | 187 |
| PMS-161 | sx3 | 0.12 | 1.58 | 1.34 | 2.33 | 236 | 229 |
| PMS-162 | sx2 | 0.70 | 2.81 | 2.09 | 2.32 | 228 | 227 |
| PMS-167 | heb3 | 0.39 | 1.94 | 1.93 | 2.33 | 230 | 229 |
| PMS-176 | hen42 | 0.44 | 1.47 | 1.78 | 2.05 | 168 | 164 |
| PMS-179 | hen12 | 0.12 | 1.68 | 1.61 | 2.18 | 246 | 256 |
| PMS-180 | hen13 | 0.22 | 1.05 | 1.66 | 2.04 | 242 | 247 |
| PMS-181 | hen11 | 0.44 | 1.84 | 2.20 | 2.71 | 251 | 262 |
| PMS-182 | hen14 | 0.77 | 2.69 | 2.20 | 3.02 | 241 | 238 |
| PMS-183 | hen15 | 0.71 | 1.67 | 2.03 | 2.58 | 249 | 258 |
| PMS-184 | hen16 | 0.27 | 0.76 | 1.75 | 1.98 | 244 | 237 |
| PMS-185 | hen17 | 0.43 | 1.58 | 1.72 | 1.67 | 239 | 251 |
| PMS-186 | hen18 | 0.26 | 1.17 | 1.74 | 1.25 | 236 | 235 |
| PMS-188 | hen19 | 0.59 | 1.64 | 1.98 | 2.46 | 244 | 248 |
| PMS-189 | hen20 | 0.38 | 4.23 | 1.95 | 2.40 | 257 | 262 |
| PMS-190 | hen21 | 0.56 | 1.45 | 2.04 | 2.53 | 247 | 251 |
| PMS-191 | hen22 | 0.36 | 1.19 | 1.73 | 2.05 | 251 | 257 |
| PMS-192 | hen23 | 0.93 | 2.86 | 2.43 | 2.85 | 240 | 256 |
| PMS-193 | hen9 | 0.46 | 0.67 | 1.27 | 1.29 | 247 | 251 |
| PMS-194 | hen10 | 0.49 | 0.64 | 1.71 | 1.75 | 256 | 256 |
| PMS-195 | hen25 | 0.40 | 0.58 | 1.63 | 1.95 | 241 | 241 |
| PMS-196 | hen27 | 0.37 | 1.21 | 1.98 | 2.75 | 239 | 240 |
| PMS-197 | hen26 | 0.23 | 1.24 | 1.89 | 2.35 | 254 | 240 |
| PMS-198 | hen24 | 0.23 | 1.47 | 1.90 | 1.75 | 235 | 227 |
| PMS-199 | hb21 | 0.62 | 6.94 | 1.80 | 2.52 | 244 | 229 |
| PMS-200 | hb22 | 0.52 | 1.70 | 1.70 | 2.08 | 246 | 244 |
| PMS-201 | hen31 | 0.53 | 1.63 | 1.79 | 2.18 | 246 | 251 |
| PMS-202 | hen30 | 0.73 | 2.65 | 1.91 | 1.90 | 253 | 255 |
| PMS-203 | hen28 | 0.49 | 0.73 | 1.61 | 2.19 | 249 | 253 |
| PMS-204 | hen29 | 0.92 | 4.03 | 1.84 | 2.50 | 241 | 256 |
| PMS-205 | hen33 | 0.19 | 0.66 | 1.82 | 2.16 | 252 | 257 |
| PMS-206 | hen32 | 0.38 | 2.23 | 1.39 | 1.73 | 244 | 258 |
| PMS-207 | hb14 | 0.45 | 1.00 | 1.84 | 1.98 | 245 | 255 |
| PMS-208 | hb15 | 0.39 | 0.95 | 1.80 | 1.94 | 246 | 255 |
| PMS-209 | hb17 | 0.48 | 1.08 | 2.30 | 2.78 | 243 | 257 |
| PMS-210 | hb13 | 2.98 | 2.98 | 2.46 | 2.90 | 241 | 243 |
| PMS-211 | hb16 | 0.56 | 7.30 | 2.16 | 3.02 | 241 | 228 |
| PMS-212 | gz23 | 0.58 | 1.35 | 1.85 | 2.09 | 263 | 280 |
| PMS-213 | gz21 | 0.79 | 1.08 | 1.56 | 1.60 | 261 | 280 |
| PMS-214 | gz22 | 0.59 | 1.17 | 1.83 | 1.85 | 268 | 289 |
| PMS-215 | gz24 | 0.69 | 3.20 | 1.91 | 1.68 | 264 | 287 |
| PMS-216 | gz15 | 0.44 | 1.15 | 1.64 | 1.73 | 264 | 279 |
| PMS-218 | gz18 | 0.47 | 1.84 | 2.60 | 2.02 | 265 | 288 |
| PMS-219 | gz16 | 2.58 | 4.77 | 2.58 | 2.37 | 266 | 288 |
| PMS-221 | gz31 | 0.66 | 3.50 | 1.92 | 1.96 | 266 | 288 |
| PMS-222 | gz27 | 0.70 | 2.19 | 2.00 | 1.88 | 268 | 288 |
| PMS-223 | gz28 | 1.16 | 1.57 | 2.00 | 2.10 | 269 | 290 |
| PMS-224 | gz32 | 1.01 | 2.30 | 1.75 | 1.93 | 273 | 292 |
| PMS-225 | gz19 | 0.80 | 0.88 | 2.10 | 2.18 | 271 | 290 |
| PMS-226 | gz13 | 0.96 | 2.11 | 1.80 | 1.61 | 271 | 290 |
| PMS-227 | gz10 | 0.92 | 2.61 | 2.15 | 1.72 | 271 | 290 |
| PMS-228 | gz14 | 1.18 | 3.43 | 2.35 | 2.05 | 271 | 288 |
| PMS-230 | gz12 | 1.19 | 3.64 | 1.98 | 2.35 | 269 | 291 |
| PMS-231 | gz11 | 0.96 | 1.45 | 1.99 | 1.90 | 271 | 294 |
| PMS-232 | gz5 | 1.87 | 2.93 | 2.15 | 2.45 | 275 | 296 |
| PMS-233 | gz6 | 0.91 | 3.90 | 2.48 | 2.30 | 277 | 298 |
| PMS-234 | gz7 | 0.67 | 2.83 | 1.90 | 1.95 | 267 | 288 |
| PMS-236 | gz8 | 0.46 | 2.16 | 1.51 | 1.50 | 273 | 287 |
| PMS-237 | gz9 | 0.41 | 1.74 | 1.64 | 2.45 | 272 | 289 |
| PMS-238 | gz2 | 0.37 | 3.03 | 1.59 | 1.68 | 272 | 292 |
| PMS-241 | gz4 | 1.00 | 5.15 | 1.64 | 2.20 | 271 | 291 |
| PMS-249 | yn10 | 2.40 | 6.87 | 1.96 | 2.44 | 283 | 309 |
| PMS-251 | yn7 | 1.75 | 6.41 | 1.84 | 1.86 | 283 | 284 |
| PMS-252 | yn12 | 0.74 | 4.20 | 1.54 | 2.57 | 284 | 275 |
| PMS-253 | yn13 | 1.22 | 3.66 | 1.36 | 1.92 | 286 | 281 |
| PMS-256 | yn14 | 0.61 | 2.50 | 1.40 | 2.15 | 284 | 276 |
| PMS-257 | yn8 | 0.40 | 2.58 | 1.20 | 1.53 | 285 | 281 |
| PMS-258 | yn9 | 0.93 | 2.74 | 1.39 | 1.92 | 284 | 280 |
| PMS-259 | yn6 | 0.84 | 1.21 | 1.23 | 0.87 | 284 | 285 |
| PMS-260 | yn15 | 1.13 | 5.89 | 2.15 | 2.36 | 282 | 287 |
| PMS-263 | yn1 | 0.22 | 0.62 | 1.37 | 1.24 | 164 | 169 |
| PMS-264 | yn2 | 0.83 | 2.85 | 1.15 | 1.73 | 281 | 278 |
| PMS-265 | yn4 | 0.76 | 2.63 | 1.82 | 2.25 | 277 | 270 |
| PMS-268 | yn5 | 0.67 | 2.95 | 1.50 | 2.35 | 285 | 281 |
| PMS-270 | yn3 | 1.21 | 8.29 | 1.50 | 2.40 | 286 | 282 |
| PMS-272 | jx21 | 0.36 | 2.33 | 1.75 | 2.20 | 259 | 269 |
| PMS-273 | jx23 | 0.46 | 2.58 | 1.64 | 1.85 | 257 | 273 |
| PMS-274 | jx24 | 0.25 | 1.07 | 1.41 | 1.70 | 259 | 276 |
| PMS-275 | jx22 | 0.37 | 2.42 | 1.84 | 2.26 | 258 | 273 |
| PMS-276 | jx19 | 0.52 | 1.77 | 2.30 | 2.66 | 262 | 277 |
| PMS-277 | jx20 | 0.28 | 1.27 | 1.57 | 2.14 | 259 | 274 |
| PMS-278 | jx8 | 0.55 | 1.82 | 1.57 | 2.38 | 262 | 277 |
| PMS-279 | jx9 | 0.75 | 3.48 | 1.96 | 2.18 | 258 | 273 |
| PMS-280 | jx7 | 0.32 | 4.86 | 1.72 | 2.33 | 258 | 273 |
| PMS-281 | jx6 | 1.24 | 5.57 | 2.15 | 2.80 | 256 | 267 |
| PMS-284 | ah1 | 0.37 | 3.40 | 1.56 | 2.23 | 256 | 266 |
| PMS-285 | ah5 | 1.68 | 6.01 | 1.77 | 2.22 | 257 | 269 |
| PMS-286 | ah9 | 0.67 | 2.69 | 2.39 | 3.05 | 254 | 259 |
| PMS-287 | ah2 | 0.95 | 3.96 | 2.16 | 2.65 | 254 | 266 |
| PMS-288 | ah8 | 1.68 | 7.97 | 2.12 | 3.00 | 255 | 249 |
| PMS-290 | ah6 | 1.19 | 3.34 | 2.33 | 2.97 | 256 | 260 |
| PMS-291 | ah7 | 1.02 | 7.11 | 2.60 | 2.84 | 257 | 272 |
| PMS-292 | js7 | 0.63 | 3.68 | 2.50 | 3.08 | 254 | 273 |
| PMS-293 | js6 | 1.21 | 6.47 | 2.36 | 2.68 | 254 | 258 |
| PMS-295 | js5 | 0.98 | 6.08 | 2.04 | 2.35 | 227 | 235 |
| PMS-297 | js1 | 0.49 | 1.58 | 1.57 | 2.12 | 251 | 262 |
| PMS-298 | js2 | 1.29 | 2.89 | 1.44 | 2.05 | 243 | 258 |
| PMS-299 | js3 | 0.39 | 0.85 | 1.71 | 2.35 | 247 | 261 |
| PMS-300 | zj7 | 0.84 | 1.95 | 1.56 | 2.40 | 254 | 254 |
| PMS-301 | zj6 | 0.23 | 2.36 | 1.42 | 2.03 | 250 | 244 |
| PMS-302 | zj5 | 0.54 | 2.01 | 1.99 | 2.55 | 256 | 265 |
| PMS-303 | zj4 | 0.45 | 3.56 | 1.95 | 2.65 | 256 | 249 |
| PMS-304 | zj3 | 0.37 | 3.30 | 2.06 | 2.50 | 253 | 263 |
| PMS-305 | zj1 | 0.56 | 1.93 | 1.81 | 2.46 | 259 | 273 |
| PMS-306 | zj2 | 0.84 | 2.41 | 1.61 | 2.67 | 259 | 275 |
| PMS-307 | zj26 | 0.12 | 1.14 | 1.79 | 2.30 | 257 | 273 |
| PMS-308 | zj25 | 0.25 | 2.02 | 1.84 | 2.45 | 256 | 251 |
| PMS-309 | zj24 | 0.21 | 1.04 | 1.42 | 1.85 | 255 | 269 |
| PMS-310 | zj8 | 0.50 | 2.62 | 1.79 | 2.42 | 255 | 269 |
| PMS-311 | zj23 | 0.55 | 2.13 | 1.94 | 2.05 | 248 | 238 |
| PMS-312 | fj1 | 0.79 | 2.89 | 1.89 | 2.27 | 268 | 289 |
| PMS-313 | fj10 | 1.31 | 3.32 | 2.30 | 2.98 | 273 | 292 |
| PMS-314 | fj11 | 1.25 | 4.08 | 2.10 | 2.70 | 265 | 281 |
| PMS-315 | fj9 | 0.93 | 2.21 | 2.10 | 2.42 | 273 | 291 |
| PMS-316 | fj8 | 0.93 | 2.37 | 2.08 | 2.98 | 272 | 291 |
| PMS-317 | fj7 | 2.02 | 3.57 | 1.75 | 2.35 | 272 | 289 |
| PMS-318 | fj6 | 0.82 | 1.83 | 1.85 | 2.18 | 271 | 281 |
| PMS-322 | fj2 | 1.41 | 3.88 | 1.95 | 2.55 | 265 | 260 |
| PMS-323 | fj3 | 1.50 | 5.81 | 2.40 | 2.72 | 271 | 289 |
| PMS-324 | fj15 | 1.80 | 3.35 | 2.03 | 1.98 | 271 | 289 |
| PMS-325 | fj14 | 1.56 | 6.29 | 2.45 | 2.89 | 272 | 290 |
| PMS-327 | fj13 | 1.45 | 5.39 | 2.05 | 2.38 | 274 | 292 |
| PMS-329 | fj16 | 1.95 | 5.00 | 2.00 | 2.65 | 276 | 290 |
| PMS-330 | fj19 | 1.10 | 1.52 | 2.10 | 2.55 | 271 | 288 |
| PMS-331 | fj18 | 1.91 | 5.67 | 2.30 | 2.52 | 276 | 285 |
| PMS-332 | fj20 | 2.97 | 4.59 | 1.70 | 2.48 | 276 | 295 |
| PMS-333 | fj21 | 4.54 | 18.35 | 2.42 | 2.83 | 273 | 290 |
| PMS-334 | fj22 | 0.77 | 2.39 | 2.06 | 2.32 | 273 | 298 |
| PMS-335 | jx13 | 0.68 | 2.75 | 2.40 | 2.80 | 268 | 288 |
| PMS-336 | jx14 | 0.77 | 2.06 | 2.35 | 1.98 | 271 | 290 |
| PMS-337 | jx15 | 0.82 | 7.71 | 1.50 | 2.72 | 271 | 290 |
| PMS-338 | jx16 | 0.77 | 2.58 | 2.00 | 2.04 | 268 | 282 |
| PMS-339 | jx17 | 1.63 | 5.55 | 1.70 | 2.06 | 265 | 282 |
| PMS-340 | jx1 | 2.23 | 6.39 | 2.25 | 2.62 | 269 | 288 |
| PMS-341 | jx4 | 2.56 | 2.62 | 1.78 | 1.75 | 265 | 285 |
| PMS-342 | jx2 | 1.15 | 3.45 | 2.40 | 2.30 | 271 | 292 |
| PMS-343 | jx5 | 0.37 | 1.05 | 1.70 | 2.30 | 271 | 289 |
| PMS-344 | jx3 | 1.08 | 5.76 | 1.87 | 1.85 | 269 | 262 |
| PMS-345 | gd15 | 1.69 | 6.41 | 2.35 | 2.85 | 271 | 264 |
| PMS-346 | gd24 | 0.73 | 2.83 | 2.00 | 2.10 | 276 | 262 |
| PMS-348 | gd25 | 3.12 | 9.05 | 2.25 | 2.28 | 273 | 266 |
| PMS-349 | gd26 | 0.90 | 5.92 | 2.25 | 2.50 | 278 | 262 |
| PMS-350 | gd23 | 1.37 | 5.94 | 2.24 | 3.06 | 279 | 269 |
| PMS-351 | gd16 | 3.36 | 38.49 | 1.82 | 3.10 | 278 | 266 |
| PMS-352 | gd18 | 2.23 | 7.24 | 1.80 | 3.00 | 279 | 273 |
| PMS-353 | gd17 | 2.06 | 7.12 | 1.05 | 2.48 | 297 | 301 |
| PMS-354 | gd6 | 5.12 | 12.59 | 1.68 | 2.52 | 278 | 295 |
| PMS-356 | gd34 | 3.65 | 15.74 | 1.64 | 2.24 | 281 | 278 |
| PMS-359 | gd28 | 1.77 | 9.56 | 2.15 | 2.39 | 274 | 263 |
| PMS-360 | gd27 | 1.10 | 7.21 | 1.86 | 2.50 | 278 | 286 |
| PMS-361 | gd19 | 0.90 | 4.87 | 1.90 | 1.60 | 278 | 272 |
| PMS-362 | gd20 | 1.41 | 8.22 | 1.67 | 2.65 | 279 | 296 |
| PMS-363 | gd22 | 1.25 | 7.06 | 1.59 | 2.56 | 275 | 266 |
| PMS-364 | gd21 | 1.49 | 5.36 | 1.72 | 2.39 | 274 | 265 |
| PMS-372 | gd11 | 2.73 | 16.57 | 1.32 | 2.43 | 295 | 289 |
| PMS-373 | gd12 | 3.13 | 8.36 | 1.90 | 3.02 | 288 | 281 |
| PMS-377 | hn3 | 4.81 | 13.37 | 1.16 | 2.30 | 295 | 296 |
| PMS-379 | hn2 | 1.82 | 12.05 | 1.15 | 1.68 | 306 | 309 |
| PMS-380 | hn1 | 2.07 | 7.74 | 1.88 | 2.12 | 360 | 360 |
| PMS-381 | hn6 | 0.64 | 1.98 | 0.90 | 2.08 | 360 | 360 |
| PMS-382 | hn5 | 1.33 | 2.04 | 0.86 | 1.63 | 360 | 360 |
| PMS-383 | hn4 | 1.29 | 2.17 | 0.61 | 1.59 | 360 | 360 |
| PMS-385 | gx23 | 2.47 | 0.39 | 2.03 | 1.68 | 279 | 273 |
| PMS-386 | gx24 | 7.89 | 16.65 | 2.26 | 3.12 | 286 | 277 |
| PMS-387 | gx25 | 2.61 | 7.91 | 1.59 | 2.33 | 283 | 284 |
| PMS-388 | gx20 | 1.37 | 7.30 | 2.12 | 3.03 | 279 | 269 |
| PMS-389 | gx22 | 2.21 | 4.39 | 2.40 | 2.98 | 279 | 275 |
| PMS-390 | gx21 | 6.51 | 9.77 | 3.00 | 2.20 | 276 | 268 |
| PMS-391 | gx17 | 4.35 | 9.60 | 2.07 | 2.68 | 280 | 280 |
| PMS-392 | gx18 | 4.43 | 11.63 | 2.32 | 3.03 | 277 | 272 |
| PMS-393 | gx19 | 4.98 | 22.18 | 2.80 | 2.99 | 276 | 271 |
| PMS-395 | gx14 | 5.43 | 12.99 | 2.90 | 3.05 | 270 | 262 |
| PMS-396 | gx16 | 3.32 | 15.56 | 2.80 | 3.26 | 270 | 262 |
| PMS-397 | gx15 | 3.27 | 10.17 | 3.10 | 3.25 | 271 | 263 |
| PMS-398 | gx13 | 3.80 | 15.29 | 2.78 | 3.20 | 270 | 262 |
| PMS-399 | gx12 | 4.49 | 8.91 | 2.37 | 3.00 | 273 | 263 |
| PMS-400 | gx11 | 3.91 | 9.26 | 2.47 | 3.05 | 276 | 263 |
| PMS-401 | gx8 | 0.89 | 7.33 | 1.98 | 3.04 | 271 | 258 |
| PMS-402 | gx9 | 1.68 | 6.85 | 2.20 | 3.25 | 271 | 262 |
| PMS-403 | gx10 | 2.29 | 3.78 | 2.15 | 2.62 | 271 | 261 |
| PMS-404 | gx7 | 1.29 | 4.54 | 2.20 | 3.12 | 276 | 264 |
| PMS-405 | hun3 | 1.21 | 1.29 | 1.70 | 2.44 | 264 | 279 |
| PMS-406 | hun2 | 1.04 | 5.64 | 1.70 | 2.60 | 271 | 288 |
| PMS-407 | hun4 | 1.39 | 9.00 | 2.05 | 2.28 | 265 | 278 |
| PMS-408 | hun1 | 3.28 | 10.72 | 1.80 | 2.72 | 265 | 280 |
| PMS-409 | hun5 | 3.17 | 5.53 | 1.79 | 2.70 | 265 | 280 |
| PMS-410 | hun30 | 3.44 | 7.04 | 2.10 | 2.82 | 265 | 280 |
| PMS-411 | hun29 | 2.19 | 5.57 | 2.18 | 2.47 | 268 | 284 |
| PMS-412 | hun28 | 1.68 | 3.36 | 1.53 | 2.45 | 267 | 285 |
| PMS-413 | hun25 | 2.89 | 5.46 | 1.89 | 2.62 | 265 | 281 |
| PMS-414 | hun26 | 1.59 | 0.99 | 1.40 | 1.50 | 264 | 280 |
| PMS-415 | hun7 | 4.18 | 6.19 | 1.90 | 2.73 | 261 | 269 |
| PMS-416 | hun6 | 1.95 | 5.20 | 1.41 | 2.42 | 260 | 272 |
| PMS-418 | hun16 | 2.92 | 9.55 | 1.80 | 2.72 | 264 | 276 |
| PMS-419 | hun14 | 3.28 | 5.71 | 1.95 | 2.80 | 257 | 272 |
| PMS-420 | hun15 | 1.04 | 2.48 | 2.05 | 2.82 | 259 | 272 |
| PMS-421 | hun20 | 1.84 | 4.37 | 1.97 | 2.45 | 264 | 279 |
| PMS-422 | hun21 | 1.20 | 6.97 | 1.98 | 2.45 | 261 | 274 |
| PMS-423 | hun22 | 2.05 | 2.91 | 1.86 | 2.52 | 256 | 269 |
| PMS-424 | hun23 | 1.59 | 6.53 | 1.71 | 2.68 | 263 | 277 |
| PMS-425 | hb6 | 0.87 | 3.46 | 1.70 | 2.45 | 258 | 273 |
| PMS-426 | hb2 | 0.28 | 1.91 | 1.89 | 2.39 | 258 | 271 |
| PMS-427 | hb3 | 0.78 | 5.20 | 1.90 | 3.06 | 257 | 269 |
| PMS-428 | hb4 | 1.55 | 4.09 | 1.80 | 3.10 | 254 | 265 |
| PMS-429 | hb5 | 1.23 | 8.29 | 1.91 | 2.85 | 258 | 273 |
| PMS-430 | hb47 | 0.77 | 2.89 | 1.85 | 2.84 | 254 | 269 |
| PMS-431 | hb46 | 0.45 | 6.06 | 2.14 | 2.95 | 255 | 267 |
| PMS-432 | hb45 | 0.96 | 2.90 | 1.62 | 2.05 | 254 | 269 |
| PMS-433 | hb44 | 1.12 | 6.41 | 1.87 | 2.30 | 254 | 259 |
| PMS-439 | usa1 | 0.78 | 2.43 | 1.15 | 1.82 | 259 | 265 |
| PMS-440 | usa2 | 0.57 | 1.60 | 1.15 | 1.70 | 256 | 267 |
| PMS-441 | hb38 | 1.38 | 4.97 | 1.52 | 2.27 | 258 | 267 |
| PMS-444 | gd1 | 0.74 | 6.97 | 1.43 | 2.36 | 285 | 279 |
| PMS-447 | gd4 | 0.48 | 2.35 | 1.36 | 2.38 | 282 | 295 |
| PMS-448 | gd5 | 0.82 | 2.58 | 1.26 | 2.38 | 282 | 300 |
| PMS-449 | gd33 | 2.24 | 7.17 | 1.46 | 2.86 | 290 | 280 |
| PMS-454 | gd32 | 1.34 | 12.02 | 1.03 | 3.10 | 294 | 299 |
| PMS-462 | sd2 | 0.80 | 1.31 | 1.55 | 1.89 | 232 | 233 |
| PMS-466 | sd1 | 0.43 | 8.10 | 1.07 | 1.96 | 236 | 227 |
| PMS-468 | sc13 | 0.47 | 12.52 | 1.41 | 2.68 | 243 | 228 |
| PMS-469 | sc45 | 0.63 | 10.73 | 1.85 | 2.87 | 264 | 273 |
| PMS-470 | sc44 | 0.58 | 9.24 | 2.05 | 3.23 | 257 | 273 |
| PMS-471 | sc8 | 2.40 | 25.93 | 2.60 | 3.05 | 263 | 280 |
| PMS-474 | sc29 | 0.86 | 10.34 | 2.30 | 2.74 | 258 | 254 |
| PMS-476 | sc33 | 0.89 | 6.67 | 2.20 | 3.00 | 258 | 257 |
| PMS-477 | sc31 | 0.96 | 6.00 | 2.24 | 2.72 | 259 | 257 |
| PMS-478 | sc32 | 1.61 | 18.53 | 1.65 | 2.89 | 259 | 273 |
| PMS-480 | jx18 | 0.87 | 10.56 | 1.50 | 2.45 | 259 | 248 |
| PMS-482 | sc3 | 0.46 | 1.25 | 1.77 | 2.92 | 266 | 280 |
