# Supplementary material for: Genetic Diversity and Population Structure of Miscanthus sinensis Germplasm in China
Source: PLoS One. 2013 Oct 7;8(10):e75672. doi: 10.1371/journal.pone.0075672 (PMC3792140; doi:10.1371/journal.pone.0075672)
Supplement: Table S4 — The genotypic data of 115 alleles in the M. sinensis accessions. (DOC) [file pone.0075672.s004.doc]

**Table S4** Primer sequences, optimal annealing temperature (AT), number of polymorphic bands (NB) and chromosome location on the *B. distachyon* for each primer pair

| Primer ^a^ | Sequence ^b^ | AT ^c^ (℃) | NB^d^ | Chromosome location^e^ |
| --- | --- | --- | --- | --- |
| DBM-28F | CAAAAGCAGCACCAAAATCAG | 50 | 6 | 3 |
| DBM-28R | TTGCTCATCGTCCATAGATCC |  |  |  |
| DBM-8F | CTCTCCATCGCCTACTTGGTC | 55 | 6 | 1 |
| DBM-8R | GTTTCTTGGATCTTGGCCTTT |  |  |  |
| DBM-15F | ATACCACCTCCACCATTACCC | 55 | 3 | 1 |
| DBM-15R | GTATTCGCCTAATGCCAGGA |  |  |  |
| DBM-38F | CTGTCCTCCCTCAGATCGAC | 55 | 5 | 3 |
| DBM-38R | CACATCCATTCCTCTCTTCCA |  |  |  |
| DBM-42F | GTCGAATATCTGGATCGAACG | 55 | 3 | 4 |
| DBM-42R | AGGCTGGCTGGAAGAAGAG |  |  |  |
| DBM-51F | ACTTCTGCTGCTGCTACCTTG | 55 | 6 | 5 |
| DBM-51R | ATGCGCAAGAGTCTAGATGGA |  |  |  |
| DBM-53F | CATCACGAGGAGCAAAAGAGT | 55 | 6 | 5 |
| DBM-53R | GAGGGGATAGGAGAGGGAGAG |  |  |  |
| DBM-4F | CAGATGAGATCCGTTCAGCTC | 60 | 4 | 1 |
| DBM-4R | ACATGTCACGGCTGGTCTC |  |  |  |
| DBM-12F | GGCAATGGCACACAAGAGATA | 60 | 16 | 1 |
| DBM-12R | CAGCTATGACATGCAAATGGA |  |  |  |
| DBM-18F | AACGTCATCGTCTTCAACACC | 60 | 2 | 2 |
| DBM-18R | TCAATGCAGTTTTGTGATGGA |  |  |  |
| DBM-22F | GCGCAAGTTAGAACTGCAATC | 60 | 1 | 2 |
| DBM-22R | TTGGACTCTTTGCTAATGCAAC |  |  |  |
| DBM-24F | GTGGCGGAAACCCTAGCC | 60 | 5 | 2 |
| DBM-24R | GCCGTGGTAGATATCCGAGA |  |  |  |
| DBM-25F | GAGATGCTCACGCTTCCAAT | 60 | 4 | 2 |
| DBM-25R | TCGAGGAACAAAATCAACTTCA |  |  |  |
| DBM-26F | CGGCTCCGATCTGCTAAG | 60 | 5 | 2 |
| DBM-26R | GAGTGACCTCGTCGGAGAAG |  |  |  |
| DBM-36F | ATCCTGGCGCTCATCCTC | 60 | 10 | 3 |
| DBM-36R | AGGAGGAGAGTGGCGTTGT |  |  |  |
| DBM-39F | CCTTGACGGCGGTAGTAACTT | 60 | 2 | 3 |
| DBM-39R | CGGAAAGGAACACCCATATTT |  |  |  |
| DBM-45F | CAGATGTGGTCGAGATGTGTG | 60 | 4 | 4 |
| DBM-45R | GCAGGTAGGTCCTCAGAATGC |  |  |  |
| DBM-49F | CTTCCGCTTGATCCCCTTG | 60 | 3 | 4 |
| DBM-49R | GTGCTCTGCTTCCTGCTGT |  |  |  |
| DBM-55F | GGAGATAGGGAATTCGGGAGT | 60 | 7 | 5 |
| DBM-55R | AGAATAGCCAACGGCGAGT |  |  |  |
| DBM-2F | ATTGTGCGAGTGTGCATTGT | 62 | 3 | 1 |
| DBM-2R | CCGTCGACTTGGCGTACC |  |  |  |
| DBM-29F | GTTGCCGTCTTCGTTGAGG | 62 | 5 | 3 |
| DBM-29R | AAGATGAAGAGCTTGCTGTCG |  |  |  |
| DBM-56F | AGAAGGTGGTGAGGTTGGAGT | 62 | 3 | 5 |
| DBM-56R | GAACAGAGCAGCAGAGCAGAG |  |  |  |
| DBM-58F | TGAATAAAATGCACTACCTGC | 62 | 6 | 5 |
| DBM-58R | AACACGATGTCCCCCTTG |  |  |  |

^a^ F, Forward primer; R, Reverse primer

^b^ Primer sequence

^c^ Optimal annealing temperature for different Primer pairs

^d^ Number of polymorphic bands in the *M*. *sinensis* accessions

^e^ The primer on the location of *Brachypodium distachyon* chromosome
